# The Near-Eastern Roots of the Neolithic in South Asia

**DOI:** 10.1371/journal.pone.0095714

**Published:** 2014-05-07

**Authors:** Kavita Gangal, Graeme R. Sarson, Anvar Shukurov

**Affiliations:** School of Mathematics and Statistics, Newcastle University, Newcastle upon Tyne, United Kingdom; University of Florence, Italy

## Abstract

The Fertile Crescent in the Near East is one of the independent origins of the Neolithic, the source from which farming and pottery-making spread across Europe from 9,000 to 6,000 years ago at an average rate of about 1 km/yr. There is also strong evidence for causal connections between the Near-Eastern Neolithic and that further east, up to the Indus Valley. The Neolithic in South Asia has been far less explored than its European counterpart, especially in terms of absolute (^14^C) dating; hence, there were no previous attempts to assess quantitatively its spread in Asia. We combine the available ^14^C data with the archaeological evidence for early Neolithic sites in South Asia to analyze the spatio-temporal continuity of the Neolithic dispersal from the Near East through the Middle East and to the Indian subcontinent. We reveal an approximately linear dependence between the age and the geodesic distance from the Near East, suggesting a systematic (but not necessarily uniform) spread at an average speed of about 0.65 km/yr.

## Introduction

The term ‘Neolithic’ was originally introduced by Sir John Lubbock in 1865 to describe the refinement in tool-making technology at the end of the Stone Age [1]. The term now has become largely synonymous with the advent of food production [2]. The Neolithic represents a set of (often related) traits, the most prominent ones being crop cultivation, animal domestication and pastoralism, pottery making, and sedentism. Although the individual traits were neither simultaneously developed nor adopted together everywhere, they do appear to have been closely linked [3]–[5].

The spread of the Neolithic in Europe was first studied quantitatively in the 1970s, when a sufficient number of ^14^C age determinations for early Neolithic sites had become available. Ammerman and Cavalli-Sforza [6] discovered a linear relationship between the age of an Early Neolithic site and its distance from the conventional source in the Near East (Jericho), thus demonstrating that, *on average,* the Neolithic spread at a *constant speed* of about 1 km/yr (see also [7]). More recent studies confirm these results and yield the speed of 0.6–1.3 km/yr at 95% confidence level [8]. This coarse-grained, large-scale picture applies at spatial scales of order hundreds of kilometers and time intervals of hundreds of years without precluding significant variations in the rate and direction of the dispersal at smaller spatio-temporal scales [9]–[11]. In particular, a ‘leap-frog’ colonization (which could be especially important in coastal and riverine areas [4], [12], [13]) involving directed, relatively rapid movements over distances of order 100 km or less is fully consistent with this global picture. Likewise, as evidenced by sufficiently realistic models [13]–[16], such a spread does not need to be unidirectional or uniform (see [17] for a review).

Here we make the first attempt to quantify the Neolithic dispersal across South Asia at the simplest level, by exploring the connection between the age *T_o_* of the advent of the Neolithic and the distance *D* from its plausible source(s) in the Near East. Such an extremely coarse-grained analysis is a necessary step before any more detailed work which would include regional variations in the speed and direction of the spread.

A difficulty inherent in any study of the spread of incipient agriculture is the identification of the time of the first appearance of the Neolithic at a given location. Even a firmly established earliest evidence of the Neolithic at an archaeological site does not necessarily correspond to the arrival of the Neolithic to the wider local area, since that site might have been occupied at a later time, rather than by the first Neolithic farmers in the region. And the earliest Neolithic layer has not always been discovered (and then dated) with confidence. This problem is less prominent in the case of the better explored European Neolithic, but becomes acute in Asia.

Thus, the earliest Neolithic dates available tell us that the Neolithic appeared in that region *not later* than the available dates suggest. In terms of the dependence of the earliest known Neolithic date *T* on the distance to the source of the dispersal *D*, this implies that all the data points must lie below the line 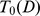, where 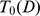 is the true (generally, unknown) arrival date at a distance *D* (assuming that earlier dates are plotted higher, as in figures shown below). In other words, the line 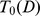 is the upper *envelope* of the data points in the (*D*, *T*) -plane: ideally, no correctly identified and accurately dated Neolithic data point can lie above this line. Earlier authors presumed that the dates available (most often, obtained after careful selection) do represent the true ‘first arrival’ time and then fitted a certain dependence 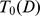. On the contrary, we explicitly allow for the fact that, even after the most careful selection, one cannot guarantee that the true arrival time to a given distance has been identified: we seek an upper envelope for the data points in the 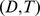-plane.

However, any age determination of an archaeological site contains random and systematic errors which most often are difficult or impossible to estimate (even in the case of ^14^C dates). These uncertainties can place a data point above the curve 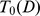. More importantly, any local acceleration of the spread can also produce a data point lying above the globally averaged dependence 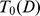, by producing a local Neolithic arrival time that is earlier than the average value of 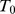 at the relevant distance *D*. Therefore, our determination of the envelope representing the globally averaged arrival time 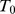 must rely on statistical procedures. We analyze a compilation of ^14^C and archaeological age determinations for the early Neolithic sites in South Asia to reveal and quantify the spatio-temporal continuity of the Neolithic dispersal in Southern Asia.

### The West-east Connection in the Asian Neolithic

There are several lines of evidence that support the idea of connection between the Neolithic in the Near East and in the Indian subcontinent. The prehistoric site of Mehrgarh in Baluchistan (modern Pakistan) is the earliest Neolithic site in the north-west Indian subcontinent, dated as early as 8500 BCE [18].

Neolithic domesticated crops in Mehrgarh include more than 90% barley and a small amount of wheat. There is good evidence for the local domestication of barley and the zebu cattle at Mehrgarh [19], [20], but the wheat varieties are suggested to be of Near-Eastern origin, as the modern distribution of wild varieties of wheat is limited to Northern Levant and Southern Turkey [21]. A detailed satellite map study of a few archaeological sites in the Baluchistan and Khybar Pakhtunkhwa regions also suggests similarities in early phases of farming with sites in Western Asia [22]. Pottery prepared by sequential slab construction, circular fire pits filled with burnt pebbles, and large granaries are common to both Mehrgarh and many Mesopotamian sites [23]. The postures of the skeletal remains in graves at Mehrgarh bear strong resemblance to those at Ali Kosh in the Zagros Mountains of southern Iran [19]. Clay figurines found in Mehrgarh resemble those discovered at Zaghe on the Qazvin plain south of the Elburz range in Iran (the 7th millennium BCE) and Jeitun in Turkmenistan (the 6th millennium BCE) [24]. Strong arguments have been made for the Near-Eastern origin of some domesticated plants and herd animals at Jeitun in Turkmenistan (pp. 225–227 in [25]).

The Near East is separated from the Indus Valley by the arid plateaus, ridges and deserts of Iran and Afghanistan, where rainfall agriculture is possible only in the foothills and *cul-de-sac* valleys [26]. Nevertheless, this area was not an insurmountable obstacle for the dispersal of the Neolithic. The route south of the Caspian sea is a part of the Silk Road, some sections of which were in use from at least 3,000 BCE, connecting Badakhshan (north-eastern Afghanistan and south-eastern Tajikistan) with Western Asia, Egypt and India [27]. Similarly, the section from Badakhshan to the Mesopotamian plains (the Great Khorasan Road) was apparently functioning by 4,000 BCE and numerous prehistoric sites are located along it, whose assemblages are dominated by the Cheshmeh-Ali (Tehran Plain) ceramic technology, forms and designs [26]. Striking similarities in figurines and pottery styles, and mud-brick shapes, between widely separated early Neolithic sites in the Zagros Mountains of north-western Iran (Jarmo and Sarab), the Deh Luran Plain in southwestern Iran (Tappeh Ali Kosh and Chogha Sefid), Susiana (Chogha Bonut and Chogha Mish), the Iranian Central Plateau (Tappeh-Sang-e Chakhmaq), and Turkmenistan (Jeitun) suggest a common incipient culture [28]. The Neolithic dispersal across South Asia plausibly involved migration of the population ([29] and [25], pp. 231–233). This possibility is also supported by Y-chromosome and mtDNA analyses [30], [31].

## Data Selection

Since only the first arrival date of the Neolithic at a site matters in the present context, we need to identify the earliest Neolithic date at each of the sites considered, for which either archaeological or radiocarbon dates are available.

We use the archaeological age determinations from Appendix A of [18] for the Indian subcontinent (the definitions of the archaeological phases are from [32]), together with archaeological records from the Middle and Near East taken from various sources. A complete date list can be found in the tables S4 and S5 in Appendix S1. For sites only dated archaeologically (i.e. in terms of archaeological stages), we use the starting date of the relevant time period in our analysis. Where both archaeological and ^14^C dates for the same site are available, we use the ^14^C data as the more precise.

We have compiled the ^14^C dates from 160 Early Neolithic sites in West and South Asia [18], [33]–[35]. Many of the ^14^C dates from the Arabian peninsula [36] are also documented in the CONTEXT database. A comprehensive list of these dates and the relevant references can be found in the tables S1 and S2 in Appendix S1. For various reasons a few dates had to be left out. A list of these dates, along with laboratory numbers and reasons for discarding them, is given in the table S3 in Appendix S1. (A histogram of this combined dataset is given in figure S1 in Supporting information S1 and the distribution of dates within each of the bins is shown in figure S2 in Supporting information S1.).

For comparison, a recent analysis of the Neolithic dispersal in Europe involved 735 sites [8]; a ^14^C database for the European Neolithic contains about 640 dates for the earliest Neolithic alone [37]. The sparsity of the data in Asia significantly complicates the analysis.

Primary sources do not always agree about the attribution of a site to the Early Neolithic. For example, a number of sites classified as Chalcolithic by their authors and then in the Context database (http://context-database.uni-koeln.de) are included into the list of Neolithic sites by Marshall [34]. We considered both attributions. We excluded all ^14^C dates marked as doubtful or cited without rating in the Context database. Likewise, we omitted the “unreliable” ^14^C dates in the list of Marshall [34], but retained those that have standard deviation in excess of 150 yr since our statistical procedures have their own ways of treating errors.

We performed our statistical analysis with and without the dates from Marshall’s list that are not classified as Neolithic in the Context database, to satisfy ourselves that the results do not change significantly. Our final results are based on the largest data set available to us, i.e., that including Marshall’s list.

Most (131) sites have multiple ^14^C dates. We identify the most plausible earliest Neolithic date(s) for each site using the following criteria (further details can be found in the figure S3 in Supporting information S1; see also [38]):

1. For a site with a single ^14^C date (29 such sites), we use this date (unless it is discarded for any other reasons, such as dubious context, questionable attribution, etc.).
2. For sites that have a statistically significant number of ^14^C dates, we applied a statistical Gaussian mixture model to isolate (where possible) a well-fit temporal cluster of the oldest dates. The dates within this cluster are then used in the subsequent analysis.
3. If a well-fit cluster cannot be identified, then the mean of those dates which lie within 350 years of the site’s oldest date are used in the analysis. (If the earliest and second earliest dates are more than 350 years apart, then only the earliest date is used.)

For criterion 2, we use the *mclust* package [39] of the R programming language, which attempts to fit the dates into up to nine separate clusters assuming a Gaussian probability distribution of the dates in each cluster. The preferred number of clusters is chosen using a Bayesian Information Criterion (BIC) which quantifies the misfit between the observed dates and the model, with a penalty for models with a larger number of parameters. For further details see figure S4 in Supporting information S1.

We performed our statistical analysis both using the relevant mean date obtained from the clustering analysis, and using the full set of individual dates within the relevant clusters, to assess the robustness of our methods. Our final results are those obtained using all individual dates within the clusters.

**Figure 1:**
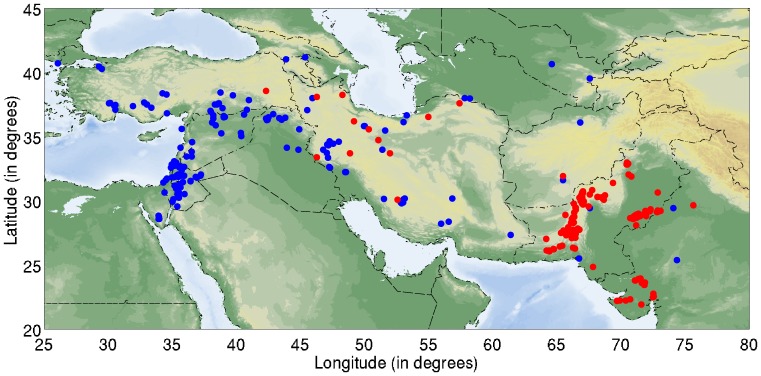
The Early Neolithic sites (10,000 BCE to 3,800 BCE) used in our analysis. Sites shown with blue symbols have ^14^C dates available, and those in red are archaeologically dated. Modern national borders are shown dashed.

Figure 1 shows the locations of the sites in our dataset, suggesting two branches in the Neolithic dispersal eastward from the Zagros: a northern route via northern Iran, southern Central Asia and Afghanistan, and a southern route via Fars through the interior of southern Iran. The emergence of the earliest Neolithic communities in Fars appears to be broadly contemporaneous with the Neolithic expansion across northern Iran [40]. It is unclear whether or not the apparent spatial gap between the two branches is an artifact of insufficient exploration. Likewise, there is a notable lack of Neolithic Jeitun-type materials in the northern Khorasan (although they possibly occur near Shahrud and Gurgan further west), which may be due to the lack of the Neolithic occupation, insufficient exploration or later alluviation [26].

## Statistical Analysis

To the best of our knowledge, there is no *suitable* standard procedure to fit an envelope *statistically* to the type of data that we have. To ensure that our results are robust, we use two distinct approaches to find an envelope that identifies the average first arrival date of the Neolithic at a given distance from a source of the spread.

We first group the data into bins according to their distance from an adopted source, such that most bins contain at least five data points (See table S1 in Supporting information S1). The bin width is also chosen to be consistent with the accuracy of the age determinations, and the expected speed of the spread. Since the *accuracy* (distinct from *precision* [41]) of the Neolithic ^14^C dates is of order 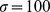
–200 yr (see the Supporting information S1) – and archaeological dates usually have larger uncertainties – and the expected average speed of the spread is 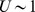 km, the width of a distance bin should be at least 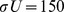 km, comparable to the width of the propagating front. We varied the bin width around this value (by considering the range 100–300 km/yr) to verify the stability of our results; as reported in the Supporting information S1, bin widths in the range 150–250 km appear to be acceptable (See figure S5 in Supporting information S1). The results presented here use a bin width of 200 km.

As in other analyses of this type, the precise position of the source of the spread is largely conventional [6]–[8], and is selected to achieve the best-quality fit to the data. We considered the six earliest Neolithic sites in the Fertile Crescent, and also all locations on a grid of 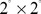 encompassing this region, and identified Gesher, one of the earliest Neolithic sites in the Jordan Valley, as the best effective source.

Having chosen the bin width and the conventional source, we consider the data distribution within each bin in two different ways, to estimate the average Neolithic arrival date 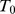 as a function of the distance *D* from the source, in terms of the linear dependence of 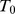 on *D*:
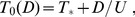
(1)where *U* is the globally averaged speed of the spread and 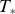 is its starting date.

**Figure 2:**
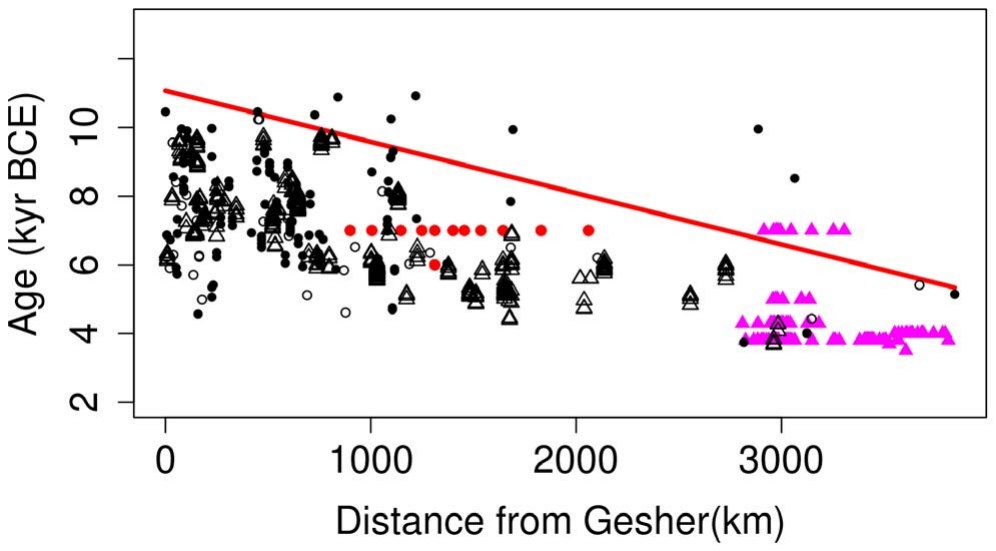
A linear envelope fit to the data using the weighted dates yields the average Neolithic dispersal speed 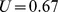 km/yr. The filled circles (red) and triangles (magenta) show the archaeologically dated sites from Iran and the Indus valley Civilization, respectively; filled circles (black) and open triangles represent sites with multiple and single ^14^C dates, respectively.

Firstly, each date 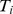 in a bin was assigned a weight 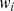, larger for the earlier dates within the bin, thus giving preference to the earlier local dates:
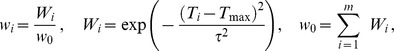
where 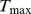 is the earliest of the 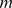 dates in the bin, 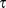 is an adjustable parameter (a weighting scale chosen empirically as described below), and the normalization factor 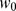 ensures that each bin carries the same weight independent of the number of dates in it. We have considered values of 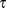 in the range 100–500 yr, to ensure that the results are robust with respect to this parameter; we present results using 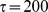
yr here (see the Supporting information S1). The best linear fit of the form (1) was then obtained using all of the data; the binning is thus only used to calculate the weights 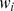. The resulting envelope, shown in Fig. 2, corresponds to 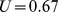 km/yr and 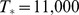 yr BCE, with the goodness of fit quantified by the coefficient of determination 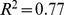 (the closer 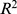 is to unity, the smaller the unexplained variance of the data point deviations from the fit).

**Figure 3:**
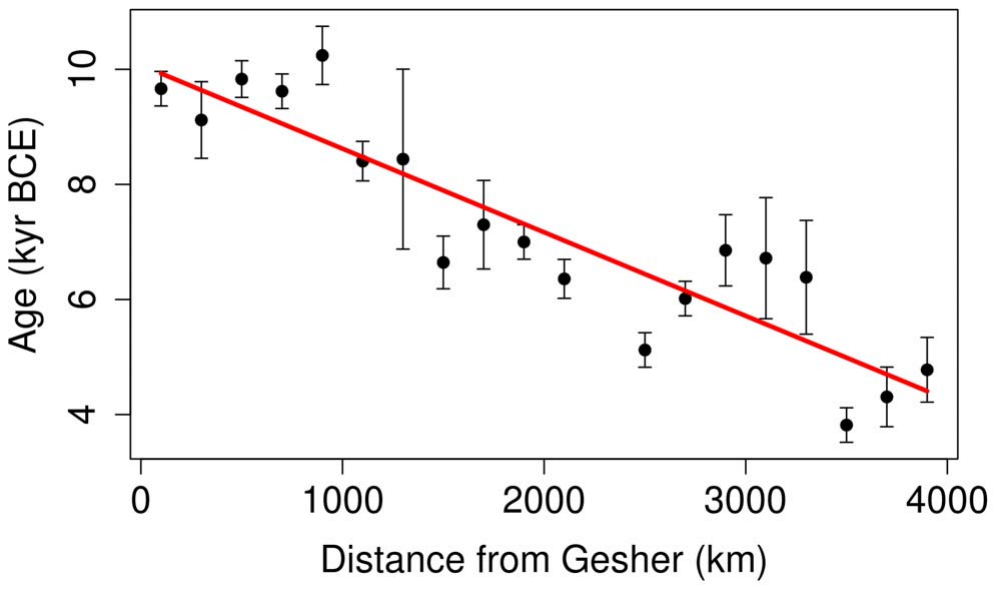
A linear envelope fit to the data using the 95-percentile points leads to a Neolithic dispersal speed 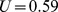 km/yr.

Secondly, a single representative first-arrival date, 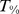, was calculated for each bin, as a certain upper percentile of the distribution of dates within the bin. Such percentiles are obtained by linear interpolation between some of the earliest dates in the bin; as such, the values are rather sensitive to the precise distribution of dates within the bin (and particularly so for bins with a small number of dates). To reduce this sensitivity, and to quantify the uncertainty in the resulting values, a bootstrapping approach was used: for each bin, the percentile value was calculated 10,000 times using sets of dates resampled randomly (with replacement) from the full set of dates in that bin; the mean of these percentile values was taken as 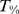 for that bin, and the standard deviation of the values was taken as the associated uncertainty, 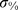. The bootstrapping procedure is described further in the Supporting information S1. Each date 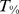 was associated with the distance *D* corresponding to the mid-distance of the corresponding bin from the source, and the best linear fit of the form (1) was obtained using 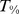 weighted by 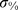, the uncertainty of this age estimate (so that less precise values of 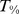 have smaller weight). We have considered various percentile levels from 70% to 97% to ensure that the results are robust in this respect, and present those for the 95% level in Fig. 3. This envelope has 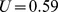 km/yr and 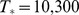
yr BCE, with 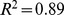. These results are close to those obtained from the weighted data as described above, lending additional confidence in the reliability of our statistical procedures.

We also considered a more complicated model that allows for a piece-wise constant dispersal speed (see figure S6 and table S2 in Supporting information S1). This model does not produce a statistically significant improvement of the results, but provides indications that the systematic spread might be better modelled as having started from a distributed source at a distance of about 1,000 km from Gesher; i.e., from the vicinity of the Zagros mountains, which appears perfectly plausible.

The values of *U* and 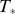 given above were obtained using the largest dataset available to us. For example, we used the individual dates within the clusters identified from our temporal clustering analysis. As noted above, however, we verified the robustness and accuracy of the results by also using, alternatively, a single representative date for each cluster. Similarly, we also used significantly modified data sets obtained by excluding the ^14^C measurements classified as Chalcolithic in the Context database. These variations in the treatment of the ^14^C data and in the data set result in values of *U* ranging from 0.58 km/yr to 0.79 km/yr, and this range is a good measure of the accuracy of our estimate of this quantity. The corresponding range of 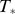 extends from 11,000 to 8,800 yr BCE.

For comparison, the 95% confidence intervals of *U* (corresponding to the 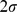 range), from the fitting procedures applied to our largest data set are 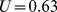
–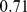 km/yr when using the weighted dates, and 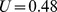
–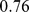 km/yr when using 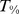 obtained as the 95%-ile. The comparable ranges for 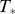 are 11,200–10,800 yr BCE and 11,200–9,400 yr BCE, respectively.

These ranges of uncertainties are affected by the difference in the number of the data points used in the two fits, however: the number of dates in the weighted method by far exceeds the number of bins in the percentile method, as is evident from comparing Figures 2 and 3. Because of this, the two methods arguably under- and over-estimate the uncertainty of the fit, respectively.

A conservative summary estimates of the average speed and starting date of the Neolithic dispersal from the Near East to the Indian Subcontinent are therefore.
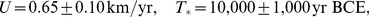
(2)where the uncertainties quoted come from comparisons of the results obtained from the various analyses mentioned above, and also from variations in the bin width, weighting scale 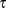 and choice of percentile-level.

## Discussion

Despite their scarcity, the ^14^C and archaeological age determinations for early Neolithic sites in Southern Asia exhibit remarkable continuity across the vast region from the Near East to the Indian Subcontinent, consistent with a systematic eastward spread at a speed of about 0.65 km/yr. It is perhaps not surprising that the rate of spread in Asia may be lower than in Europe, 1 km/yr. Firstly, the arid climate and complicated topography of the Middle East are less favorable for agriculture. Because of this, the early Neolithic settlements in Iran apparently were relatively small and widely separated. (On the other hand, the stronger reliance of the Neolithic population on herding in arid, mountainous areas, with ensuing long-distance seasonal movements, might enhance the population mobility.) Secondly, the advancement of the Neolithic in Europe was facilitated by accelerated propagation along the major European rivers (first of all, the Danube and Rhine) and the Mediterranean coastline [12], [13]. There are no major rivers in Iran and Afghanistan that could play a similar role; and the southern coastline of Iran is more arid than the country’s interior (because of the predominant northerly winds), so that the known Neolithic sites in Iran avoid the southern coastal area.

The model of the Neolithic dispersal suggested here applies at the largest, global spatial and temporal scales, as it assumes that the spread proceeded at the same speed in all directions irrespective of the local environment. Given the obvious simplicity of this model, its success in capturing the salient features of the data is encouraging. This does not diminish the need for a more detailed analysis with allowance for the local environment and palaeoclimate; but our results provide important justification, and a basis, for more sophisticated mathematical modelling.

Dispersal concepts summarily labeled as ‘wave of advance’ models, similar to that considered here, are often claimed to exclude directed individual movements and to be inconsistent with a ‘leap-frog’ colonization such as that along major waterways. In fact, both these effects, together with many other realistic refinements, can easily be included into the models without changing their conceptual and mathematical nature. Our discussion of the Neolithic dispersal can apply to demic diffusion, cultural transmission or a combination of the two. These processes only differ in the mechanisms and efficiency (speed) of the spread, but their mathematical models and spatio-temporal manifestations are closely related and only differ in details [42].

## Supporting Information

Supporting Information S1 **Supporting figures S1–S6.**
(PDF)

Appendix S1 **Supporting tables S1–S5.**
(PDF)
