# Supplementary material for: The Near-Eastern Roots of the Neolithic in South Asia
Source: PLoS One. 2014 May 7;9(5):e95714. doi: 10.1371/journal.pone.0095714 (PMC4012948; doi:10.1371/journal.pone.0095714)
Supplement: Supporting Information S1 — Supporting figures S1–S6. (PDF) [file pone.0095714.s001.pdf]

The Near-Eastern roots of the Neolithic in South Asia

Supporting information

Kavita Gangal, Graeme R. Sarson and Anvar Shukurov

School of Mathematics and Statistics, Newcastle University,
Newcastle upon Tyne, NE1 7RU, United Kingdom

This supporting information contains details of the mathematical and statistical procedures used to derive the results presented in the main text. We have made every effort to make the text accessible to non-mathematicians.

S1. Data selection and treatment

Unlike earlier quantitative studies of the Neolithic dispersal, we use both ^{14}C and (recent) archaeological age determinations in our analysis, since the number of ^{14}C dates available for Southern Asia is only modest whereas the reliability of absolute archaeological age determinations has improved significantly in recent decades. The data used is presented in the separate Appendix file to this Supporting Information. The ^{14}C dates presented in Appendix: table S1 come from the web-based database Context (http://context-database.uni-koeln.de) and from original publications referred to in the table. The absolute archaeological dates for the Indus Valley are presented in Appendix: table S4, having been conveniently compiled in [1]. The absolute archaeological dates for Iran and Afghanistan have been compiled from the various sources referred to in Appendix: table S5. Whenever both archaeological and ^{14}C dates are available for a site, we used the latter as potentially more accurate.

As discussed in the main text, the first step in our analysis was to group the data into bins according to their distance from a source point in the Jordan Valley, chosen to give the best statistical quality of the resulting analysis. The precise location of the source (Gesher in our case) is largely a technical matter (although it is important that it is within the area of the earliest Neolithic sites in the Near East), and should not be over-interpreted as corresponding to an actual prehistoric point source. As discussed in the main text, the bin width ΔD was chosen to ensure that most of the bins contain no fewer than 5 data points; table S1 suggests ΔD in the range 150–250 km is a reasonable bin width, and this range is confirmed as acceptable by the line fits obtained with varying ΔD, given in section S3.1, below. The bin width used for the rest of this work is $\Delta D = 200\,\mathrm{km}$, and the distribution of the sites between the bins is shown in Fig. S1.

Here and elsewhere in the paper, BCE dates are treated as negative: for example, for $T_1 = 10,000\,\mathrm{BCE}$ and $T_2 = 8,000\,\mathrm{BCE}$, we have $T_1 < T_2$ as $-10,000 < -8,000$. (Our figures nevertheless plot BCE ages on the positive y-axis.)

Since some bins contain a relatively small number of sites, it is essential to ensure that outliers do not affect the result.

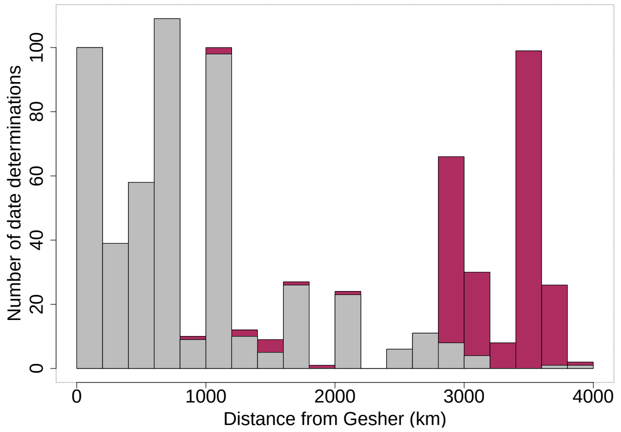

Figure S1: The histogram of the number of date determinations per distance bin, of width $\Delta D = 200\,\mathrm{km}$, with respect to the source at Gesher. The maroon shading represent archaeological dates belonging to the Neolithic, whereas the ^{14}C Neolithic dates are shown with gray shading.

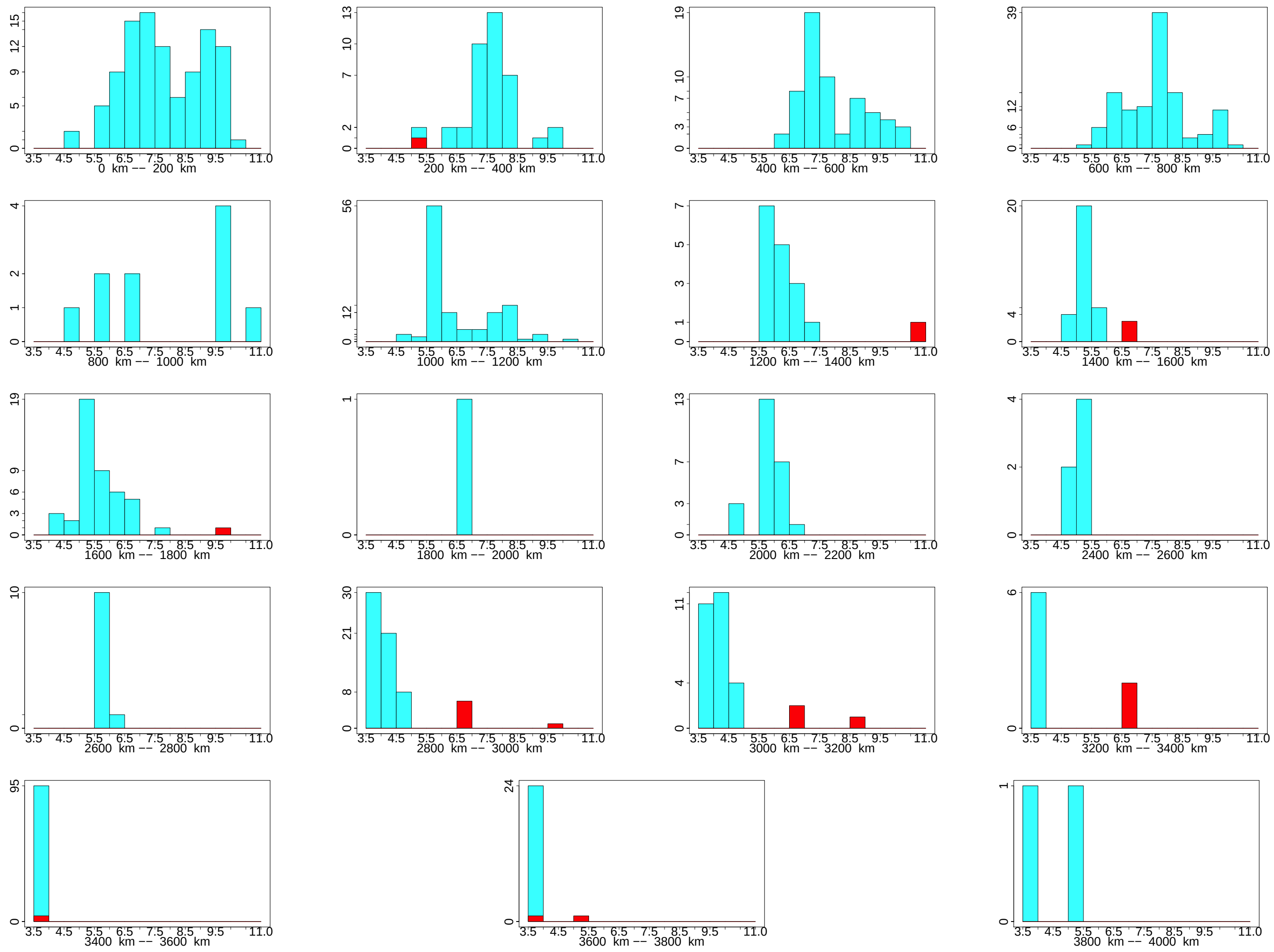

Figure S2: The distribution of the age determinations within each bin, of width $\Delta D = 200\,\text{km}$. Bin 12 (2,200–2,400 km) is empty. In each of these figures, the x-axis is in 'kyrBCE' and y-axis is the number of sites. The outliers are identified as described in the text, and are shaded in red.

Table S1: The number of data points per bin for various bin widths

Bin width (km)	Total number of bins	Number (%) of bins with more than 5 data points
100	40	28 (70%)
150	26	21 (81%)
200	20	17 (85%)
250	16	14 (87%)
300	13	12 (92%)

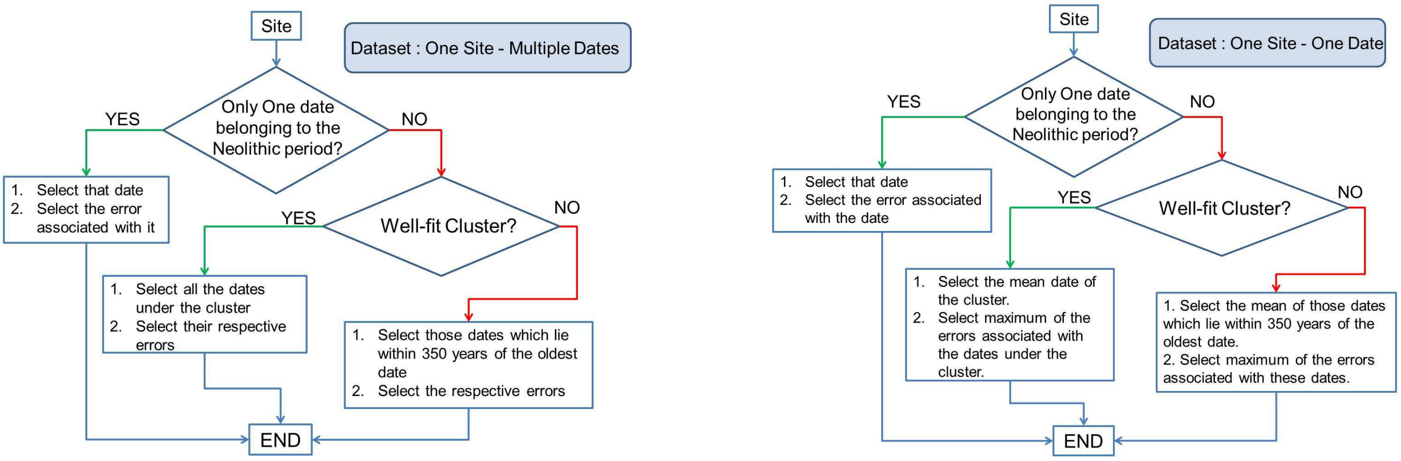

Figure S3: A flowchart of the selection procedure for the earliest Neolithic ^{14}C date(s) at a site. (a) One site, multiple dates; (b) One site, one date. See the text for further details.

The outliers, shown in Fig. S2, were identified using the interquartile ranges [34]: with Q_1 and Q_3 being the first and third quartiles of the distribution of dates with a bin (i.e., 25% of the dates in the bin are earlier than the date Q_1 and 25% of the dates are later than Q_3), a date T is treated as an outlier if it is separated from the nearest quartile by more than three interquartile ranges; i.e., if

$$T < \mathrm{Q}_1 - 3(\mathrm{Q}_3 - \mathrm{Q}_1) \quad \text{or} \quad T > \mathrm{Q}_3 + 3(\mathrm{Q}_3 - \mathrm{Q}_1).$$

Twenty five dates are classified as outliers according to this criterion. The change in the results obtained after the removal of these 25 outlier dates is not significant. Therefore, results presented in this work were obtained without discarding these outliers.

S2. The earliest Neolithic dates

Many sites have multiple ^{14}C date determinations and their treatment depends on the nature of the site and the dates (see also [35]). If there are reasons to believe that a group of dates are contemporary and only differ because of random errors, then all such dates can be used towards the analysis. We used a statistical clustering analysis to isolate, where possible, a distinct group (cluster) of the earliest Neolithic dates (using the 'mclust' Gaussian mixture model of R, as described in the main text). A similar treatment has been incorporated into OxCal 4.2 (http://c14.arch.ox.ac.uk/embed.php?File=oxcal.html) [40] as a part of the grouping analysis [41], in particular where a group of events within a sequence that can be considered as randomly sampled from a Gaussian distribution (the `Sigma_Boundary` option of OxCal). Where the earliest dates were not well fit by a Gaussian mode, we considered instead the group of all dates lying within 350 years of the oldest date. (Where no other dates are within 350 years of the oldest date, the earliest date itself is simply taken.) Then we either used all individual dates and uncertainties within these earliest groups in our analysis (as described in Fig. S3a), or used for each group a representative date and uncertainty obtained from the cluster/group analysis (as described in Fig. S3b).

To verify the stability of our results under modifications of the data set, we performed our analysis using the single representative values for the sites with multiple ^{14}C dates (i.e., based on Fig. S3b : 'One Site-One Date'; see also Appendix: table S2). Another variation of the data set, used for the same purpose, was to include or exclude the dates provided in [42] where the attribution of some ^{14}C dates as Neolithic is more inclusive than that in [3]. The variation in the final fits resulting from these variations in the data sets was used to estimate the uncertainty of our results, as presented in the main text. However, our main results were obtained with the largest data set available (i.e., including the dates from [42], and without replacing groups of dates by their representative averages; i.e., based on figure S3a).

The Gaussian mixture model was accepted for the earliest group when the following criteria are satisfied:

1. The earliest group (cluster) contains three or more dates.

2. The standard deviation of the dates in the earliest cluster, σ_1, does not exceed $\sigma_{\min} = 175\,\mathrm{yr}$, a typical accuracy of the Neolithic ^{14}C dates in our sample estimated below.

3. There is no overlap between the earliest and second earliest clusters within their standard deviations (half-widths). In other words, the two probability density functions do not cross within the ranges $T_1 \pm \sigma_1$ and $T_2 \pm \sigma_2$, where

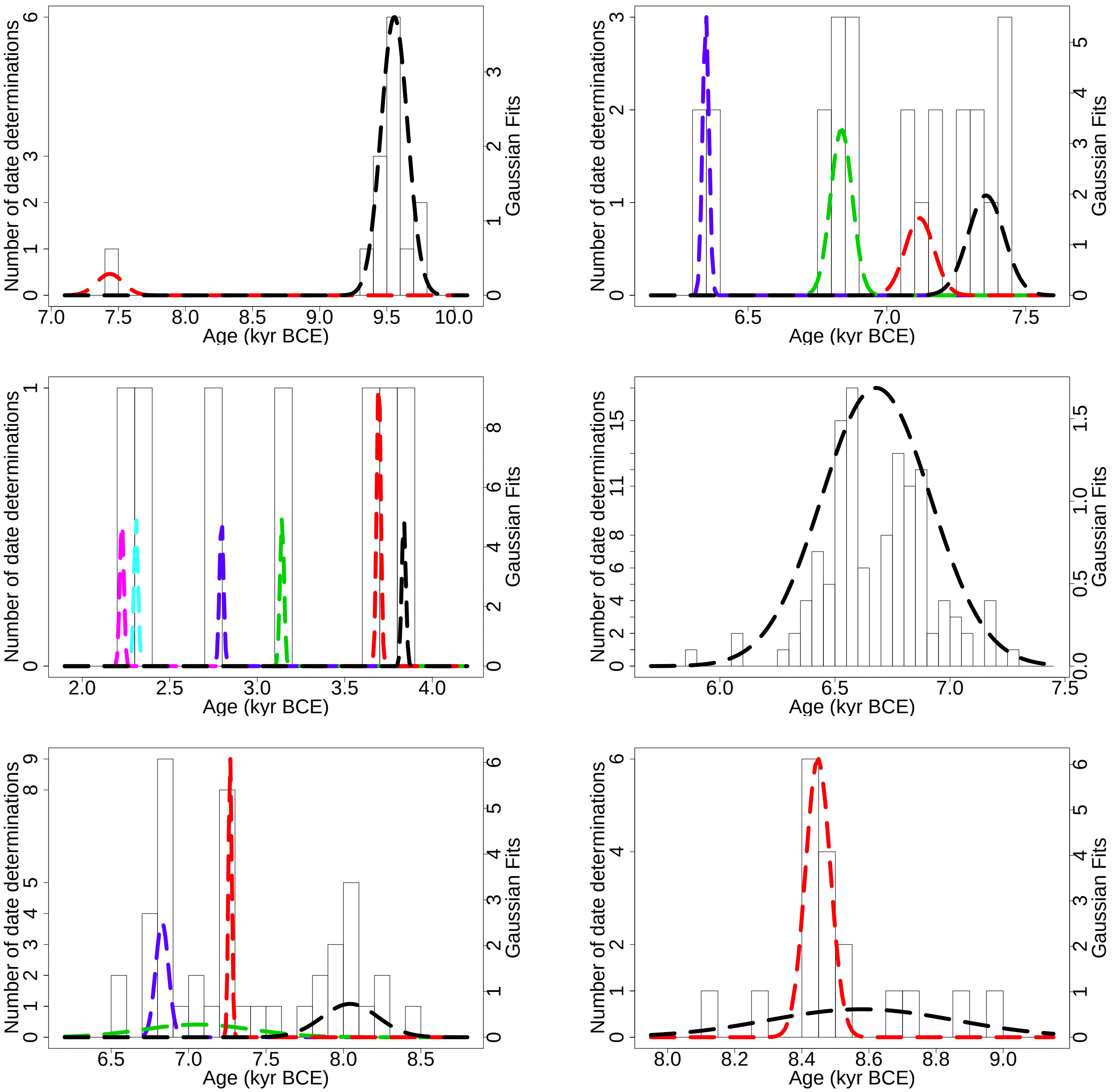

Figure S4: An illustration of the various situations encountered when calculating the earliest Neolithic date for sites with multiple ^{14}C date determinations, showing the histograms of the dates and the Gaussian-mixture fits to them (using the `mclust` routine of R) for each of the following sites: **(a: top left)** Körtik Tepe, **(b: top right)** Bouqras, **(c: middle left)** Sarazm, **(d: middle right)** Çatalhöyük East, **(e: bottom left)** Áin Ghazal and **(f: bottom right)** Djade. The Gaussian curves shown with a dashed black line in (a) and (b) represent well-defined earliest clusters according to criteria (1)–(3) of Section S2; dashed curves in other colors show later clusters. In Panels (c)–(f), results of the Gaussian mixture modeling are not acceptable.

T_1 and T_2 are the the mean dates of the earliest and second earliest clusters, respectively, and σ_1 and σ_2 are the corresponding standard deviations.

The clustering analysis is illustrated in Fig. S4 for six sites selected to represent the range of situations encountered. Each dashed curve in these figures represents one cluster (i.e., one Gaussian mode). Panels (a) and (b) show well-defined clusters; the former has no significant overlap between the earliest (black dashed curve) and second earliest (red dashed curve) groups, the latter has some overlap, but the separation of the maxima is wider than the sum of the two standard deviations. For sites with a similarly strong clustering, we show the cluster mean date and standard deviation in Appendix: table S2 and label that entry with 'C' in Column 2.

The dates shown in Panels (c)–(f) do not have well-defined clusters of the earliest dates according to the above criteria, and the Gaussian mixture model is not used in such cases. In Panel (c), the earliest 'cluster' contains a single ^{14}C date (fewer than the minimum of three dates). In Panels (d) and (e), the standard deviation of the dates in the cluster exceeds $\sigma_{\min} = 175\,\mathrm{yr}$. In Panel (f), the two clusters overlap.

In such cases, where a distinct earliest cluster of dates cannot be isolated, we use the average of all those dates which lie within 350 years ($= 2\sigma_{\min}$) of the earliest date at that site. The resulting average value and the standard deviation are shown in Appendix: table S2, labeled 'A'. If the gap between the two earliest ^{14}C dates exceeds 350 years, we simply the earliest date available, labeled 'O'.

The Boundary facility of OxCal might be also used to derive the probable date of the appearance of the Neolithic at a given site or at a certain distance from the source (i.e., within a distance bin). Unfortunately, the data available in this work are too scarce to warrant this approach.

S2.1. Data uncertainty

The published precision of the ^{14}C dates represents not their full accuracy but rather the error in the laboratory measurement of the ^{14}C content in the sample [34–36]. The calibration error alone amounts to at least 50–150 yr for the Early Neolithic period. In a specially designed experiment, Mazurkevich et al. [37] (see also [38]) obtained 35 ^{14}C dates for wooden structures belonging to four Late Neolithic dwellings in the Serteya Valley (Smolensk region, Russia) apparently constructed during a single season, so that there is every reason to expect all the dates to be contemporaneous in the sense of radiocarbon dating. The empirical standard deviation of the calibrated dates for the dwellings are 113, 83, 129 and 184 yr. Thus, the accuracy of the ^{14}C age determination is 100–200 yr in this case.

Dolukhanov et al. [35] suggest that $\sigma_{\min} = 100$–$130\,\mathrm{yr}$ for early Neolithic sites in Central and Eastern Europe. Careful inspection of the ^{14}C dates in our sample suggests that $\sigma_{\min} = 175\,\mathrm{yr}$ can be adopted as the minimum uncertainty of the ^{14}C dates for this work. We note that underestimated errors are more problematic for statistical analyses than overestimated ones. We therefore take

$$\sigma_i = \max(\tilde{\sigma}_i, 175\,\mathrm{yr}),$$

where $\tilde{\sigma}_i$ includes the laboratory error (as published together with uncalibrated ^{14}C dates) and the calibration error.

For archaeological dates, the uncertainty σ_i is taken to be the time span of the relevant archaeological stage. This gives uncertainties of up to 300 yr.

In fact, for the envelope fitting method using weightings based on the relative age within each bin (described in the main text, and below), the weighting associated with the individual date uncertainties is rather unimportant, compared to the imposed weighting. Calculations combining both sources of weighting have been made, but the results do not differ significantly from those using only the imposed weightings. For simplicity, in the present work we present only the results from the latter case.

For the envelope fitting method using percentile values for each bin, the uncertainty associated with the calculation of the percentile ($\sigma_{\%}$) is typically greater than the individual dating uncertainties, and so the former value is normally used as the uncertainty σ_i for bin i. As mentioned in the main text, the uncertainty $\sigma_{\%}$ (along with the best estimate of the percentile value, $T_{\%}$) is estimated by bootstrapping (resampling). For each bin, 10,000 synthetic data sets were created by choosing m dates at random from the full set of m dates within the bin. This is resampling with replacement, so in each synthetic set a random fraction of the original dates are replaced by duplicated original dates. (On average, this fraction will be 37% [54].) The percentile value for each synthetic set is calculated; the mean of these values is taken as $T_{\%}$, and the standard deviation as $\sigma_{\%}$. For some bins, including those where the oldest dates are multiple archaeological dates from the same stage, assigned identical dates, this procedure can give rather small values of $\sigma_{\%}$ (since the synthetic sets very often still include multiple identical earliest dates, and the relatively high percentile value becomes exactly this date). As such small uncertainties are not sensible, given that all of the individual dates going into the calculation are only known to some accuracy σ_i (for date i), the value of $\sigma_{\%}$ is taken to be a minimum of 300 yr (from the maximum archaeological dating uncertainty); i.e., the uncertainty for bin i is taken as $\sigma_i = \max(\sigma_{\%}, 300\,\mathrm{yr})$.

Similar results were obtained using alternative bootstrapping procedures (where a fixed fraction of the dates within each bin were replaced in each synthetic data set), and using jackknife methods (where synthetic data sets were created by removing a fraction of the dates within each bin, without replacement).

S3. The envelope of the data points in the (T, D)-plane

A propagating front of a spreading population (or of any other diffusing quantity) is the location where the population density (or other relevant density) reaches a constant value, arbitrary but much smaller than the maximum density far behind the front. In systems controlled by the reaction-diffusion equation, a popular model in a wide range of population dynamics applications, fronts propagate at a constant speed. Spread at a constant speed has been identified as a salient feature of the Neolithic dispersal in Europe, which suggests that various modifications of the reaction-diffusion equation and the properties of its solutions provide a suitable basis for the mathematical modeling of the Neolithic dispersal. It is therefore natural to expect that the large-scale spatio-temporal features of the spread of the Neolithic in Asia can also be modeled assuming a constant speed U:

$$T_0(D) = T_* + D/U, \tag{S1}$$

where $T_0(D)$ is the time of the first appearance of the Neolithic at a distance D from the source of the spread and T_* is the time when the spread started. This dependence can be fitted to the data to determine U and T_* using standard techniques: we largely focus on the weighted least squares method, although the (closely related) maximum likelihood method is briefly discussed below. We also consider a refinement of this model where the propagation speed is equal to different constants in two separate ranges of D to allow, to some extent, for a change in the environment with distance from the Near East.

With the weighted least squares method, the best-fitting values of U and T_* are those that provide a minimum, $\widehat{X}^2$, with respect to U and T_*, of the sum of squares of the deviations of the model of Eq. (S1) from the data T_i weighted with the uncertainties σ_i; i.e.,

$$X^2 = \sum_{i=1}^{n} \left[\frac{T_i - T_0(D_i)}{\sigma_i}\right]^2, \quad \widehat{X}^2 = \min_{(U,T_*)} X^2, \tag{S2}$$

where n is the number of the data points. $T_0(D_i)$ is the model first arrival time calculated from Eq. (S1) at the site i, which is at distance D_i from the source and has the observed arrival time T_i. As discussed in the main text, we consider fits of the form (S1) to either weighted data or to the 95%-iles of the binned data. When the weighted data are being used, in Eq. (S2) by $w_i[T_i - T_0(D_i)]^2$, with w_i defined in the main text.

The statistical quality of the fit can be conveniently quantified in terms of the coefficient of determination

$$R^2 = 1 - \frac{\widehat{X}^2}{S^2},$$

where S^2 is a measure of the deviation of the data from their overall mean value $\overline{T}$:

$$S^2 = \sum_{i=1}^{n} \left(\frac{T_i - \overline{T}}{\sigma_i}\right)^2,$$

with (see, e.g., [39])

$$\overline{T} = \frac{\sum_{i=1}^{n} T_i/\sigma_i^2}{\sum_{i=1}^{n} 1/\sigma_i^2}.$$

The coefficient of determination represents the fraction of the data variability that is accounted for by the model: $R^2 = 1$ indicates that the entirety of the scatter of the data points is explained by the model (i.e., the fit is perfect).

The weighted least-squares method implicitly assumes that the data points follow the statistical model $T_i = T_0(D_i) + \epsilon_i$, where $T_0(D) = T_* + D/U$ is our fitting function, and the errors ϵ_i are drawn from normal distributions with variance σ_i^2. For our percentile method, the root mean square value of σ_i (as obtained by bootstrapping) is 640 yr whereas the root mean square residual from our best constant speed fit (as presented in the main paper) is 990 yr. So the uncertainties in the calculation of the percentile values do not fully explain the residuals between the data and our fit. This is not particularly surprising, or concerning, since we know that our model is highly simplified, and expect deviations arising, for example, from regional variations in the local speed of spread.

An alternative formalism which explicitly includes this anticipated misfit assumes that the data points follow the statistical model $T_i = T_0(D_i) + \epsilon_i + e_i$, where ϵ_i is as above, and e_i is an additional term quantifying the additional uncertainty in our model, drawn from a normal distribution with variance σ^2; here σ is a global parameter of this model. This formalism is used in the maximum likelihood approach, which seeks the best fit by maximising the likelihood of the fit (i.e., the likelihood of the fit parameters, given the data); or equivalently, maximising the log of the likelihood, given for this model by

$$L = -\frac{1}{2}\sum_{i=1}^{n} \ln \bar{\sigma}_i^2 - \frac{1}{2}\sum_{i=1}^{n} \frac{[T_i - T_0(D_i)]^2}{\bar{\sigma}_i^2}, \quad \widehat{L} = \max_{(U,T_*,\sigma)} L,$$

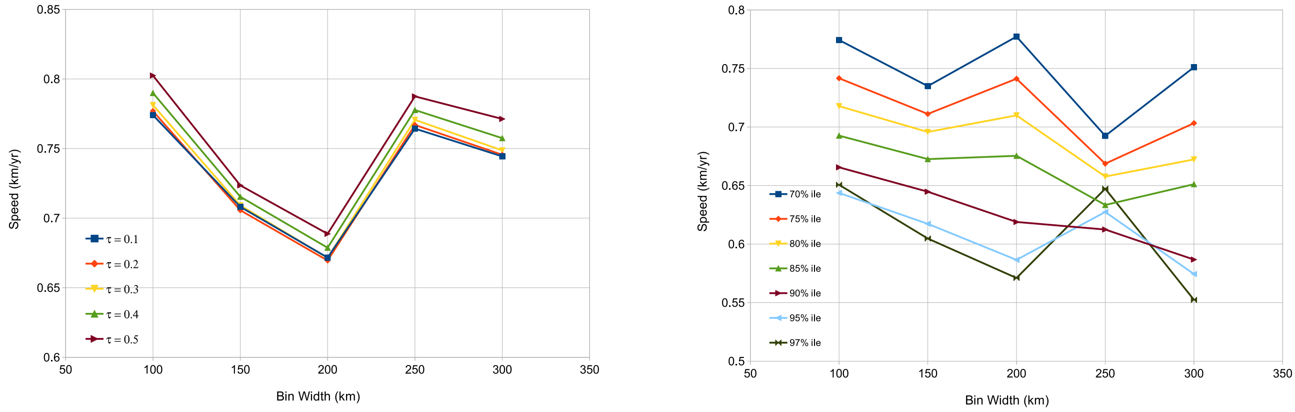

Figure S5: The variation in the best-fit speeds of spread U with the width of distance intervals used to bin the data: **(a)** for T_i taken as the weighted data, with different lines obtained for the different weighting time-scales τ, as specified in the key; **(b)** for T_i obtained as a percentile value in each bin, with different lines corresponding to the different percentile levels specified in the key.

where $\bar{\sigma}_i^2 = \sigma_i^2 + \sigma^2$. In the case of equal uncertainties σ_i, this formalism gives the same fits as the weighted least squares formalism. Applying this method to our percentile data with the σ_i obtained from bootstrapping gives a maximum likelihood estimate of σ as 610 yr, and we note that distribution tests suggest that the residuals are well modelled as coming from a normal distribution. The values of U and T_* for this fit do not differ significantly from those for the weighted least squares method, and for simplicity we present only the latter in this work.

S3.1. Constant-speed fits

The simplest model is that with a constant U, as presented above. The two methods of fits considered — using relative weightings based on the relative age within each bin, and using percentile values for each bin — were applied to this model as described in the main paper. The best fits obtained for the two methods — using bin width $\Delta D = 200\,\text{km}$, $\tau = 200\,\text{yr}$ for the weighting method and the 95%-ile value for the percentile method— were presented there. Here we present plots showing the relative insensitivity of the fits to the choices of bin width, weighting timescale and percentile value.

Figure S5a shows that U varies by only $0.1\,\text{km/yr}$ as ΔD changes from 150 to 250 km. Likewise, the results depend on τ very weakly. Figure S5b shows that percentile levels between 85 and 97%, and bin widths in the range 150–250 km, produce a similarly small variation in the values of U.

The effect of variations of ΔD, τ and percentile level on the intercept of the fits, T_*, is similarly mild.

S3.2. Variable-speed fits

There are several reasons to consider models with variable U. We are really interested here in the spread of the Neolithic beyond the Fertile Crescent, where a noticeable fraction of our ^{14}C dates belong. These dates are useful in determining the starting date of the spread, but not its speed throughout most of South Asia. Furthermore, the Zagros Mountains separate regions with very different climate and topography, and this may have affected the Neolithic dispersal. Therefore, we also considered a model which allows for a change in U at a certain distance D_0 from the source. (The choice of D_0 is described below.) Given the scarcity of the data available, we only considered the simplest models of this type, where U is constant on either side of D_0.

Instead of Eq. (S1), we thus consider the following model (similar to that of [49]):

$$T_0 = \begin{cases} T_{*1} + D/U_1 & \text{if } D < D_0, \\ T_{*2} + D/U_2 & \text{if } D > D_0, \end{cases} \tag{S3}$$

with parameters T_{*1}, U_1, T_{*2}, U_2 and D_0. Here U_1 and U_2 are the speeds of dispersal west and east of the Zagros (or any other transition region), respectively. This model allows for the dependence of T_0 on D to be discontinuous or continuous at $D = D_0$. (The discontinous fit allows for a hiatus in the spread at D_0.) In the continuous case, $T_{*2} = T_{*1} + D_0(U_1^{-1} - U_2^{-1})$ and the model has one fewer independent parameter. T_{*1} and T_{*2} are the times of the start

Table S2: Best-fit parameters and fit statistics for models with constant and variable speed of spread.

Fitted parameter	Dimension	Constant speed	Variable speed	
			Continuous fit	Discontinuous fit
U or U_1 (U_2)	km/yr	0.59	0.53 (0.84)	−2.5 (0.68)
D_0	km	–	2500	1100
T_* or T_{*1} (T_{*2})	kyr BCE	10.3	10.5 (8.7)	9.5 (9.6)
T_{*0}	kyr BCE	–	5.8	8.0
$\mathrm{AIC_c}$		24.6	29.2	25.8

of the spread projected to the source, $D = 0$. In the case of T_{*2}, this is outside the range of relevance of the second part of the fit, so it may be more useful to consider the time T_{*0} when the Neolithic started spreading at speed $U = U_2$ (i.e. for $D > D_0$); this is $T_{*0} = T_{*2} + D_0/U_2$. This quantity is also given in the Table S2.

For simplicity, we only consider these models with the data using percentile values for each bin. From the results for constant-speed fits, we do not expect the outcome using weightings based on the relative age within each bin to differ significantly.

To identify whether or not the models with variable speed are an improvement on the simpler constant-speed fit — and also to choose the best value of D_0 for the variable-speed models — we use a form of Information Criterion ([46, 48]): the second-order ('corrected') Akaike Information Criterion, $\mathrm{AIC_c}$. This criterion is more appropriate for small data sets than the first-order Akaike Information Criterion, or the Bayesian Information Criterion ([47, 48]); it is therefore more suitable for our fits, with a relatively small number of bins. It is given by

$$\mathrm{AIC_c} = n \ln\left(\widehat{X}^2/n\right) + \frac{2pn}{n-p-1} \ , \tag{S4}$$

where p is the number of free parameters in the model: $p = 2$ for the constant-speed fit (T_*, U); $p = 4$ for the continous variable-speed fit ($T_{*,1}$, U_1, U_2, D_0); $p = 5$ for the discontinous variable-speed fit ($T_{*,1}$, $T_{*,2}$, U_1, U_2, D_0). This defintion of $\mathrm{AIC_c}$ is formulated so that a smaller value of $\mathrm{AIC_c}$ corresponds to a better model. The second term in $\mathrm{AIC_c}$ acts to penalize models with more free parameters, which should of course be better able to fit the data; a more complicated model is warranted only if it provides a smaller value for the Information Criteria (i.e., if the decrease in $\widehat{X}^2$ exceeds the increase in the penalty term). In addition to $\mathrm{AIC_c}$, we have repeated the analysis using a Bayesian Information Criteria (BIC) [48] — which differs only in the relative size of the penalty term — and obtain similar conclusions.

For both type of variable-speed model (continuous or discontinuous), we varied D_0 within the range 900, 1100, . . . , 3300 km, and chose the value that minimises $\mathrm{AIC_c}$. (Within each type of model the number of free parameters does not change, so this is simply equivalent to choosing the D_0 that minimises $\widehat{X}^2$.) The best models of each type are shown in Table S2 and Fig. S6.

Using $\mathrm{AIC_c}$ to compare between the different types of models, the best model is the constant-speed model with $U = 0.59$ km/yr and $T_* = 10,283$ yr BCE, which has $\mathrm{AIC_c} = 24.6$. The discontinuous fit with $D_0 = 1100$ km is a close second, with $\mathrm{AIC_c} = 25.8$ (see table S2). Interestingly, this fit would be preferred over the constant-speed fit on the basis of the corresponding BIC values; the $\mathrm{AIC_c}$ value is the more appropriate for our small number of bins, but it may be that additional data (allowing us to use a larger number of bins) might lead to a variable-speed fit being preferred. For this discontinuous fit, the speed U at $D < D_0$ is negative, formally corresponding to spread towards the supposed source of Gesher; this part of the fit is just an artefact of our simple model based on a point source, when an extended source in the Fertile Crescent would be more realistic. But excluding the fit for $D < D_0$, this fit suggests that an extended source at $D_0 \approx 1000$ km from Gesher may be a good effective source, and the fitted speed $U_2 = 0.68$ km/yr may be a good measure of the spread to the East of the Zagros mountains. A model explicitly using an extended source merits future study. For the present work, however, we must favour the simple constant-speed fit.

In terms of the maximum likelihood interpretation of the fitting, an appropriate definition of $\mathrm{AIC_c}$ (incorporating the second order correction, and differing from the definitions in [46, 49] to follow the sense convention adopted by [48]), is

$$\mathrm{AIC_c} = -\widehat{L} + \frac{pn}{n-p-1} \ ,$$

Note that, in the context of maximum likelihood fits, σ is considered as a parameter of the model, and the $\mathrm{AIC_c}$ value for the constant-speed case would normally be calculated with $p = 3$ (U, T_*, σ). (Similarly, the continuous and discontinuous variable-speed fits would use $p = 5$ and $p = 6$. Of course, this difference in the definition of p amounts only to a constant factor, and so does not affect the comparison of $\mathrm{AIC_c}$ values between the different models considered.) Comparisons of the $\mathrm{AIC_c}$ values (and also the corresponding BIC values) for the best constant-speed and variable-speed fits obtained

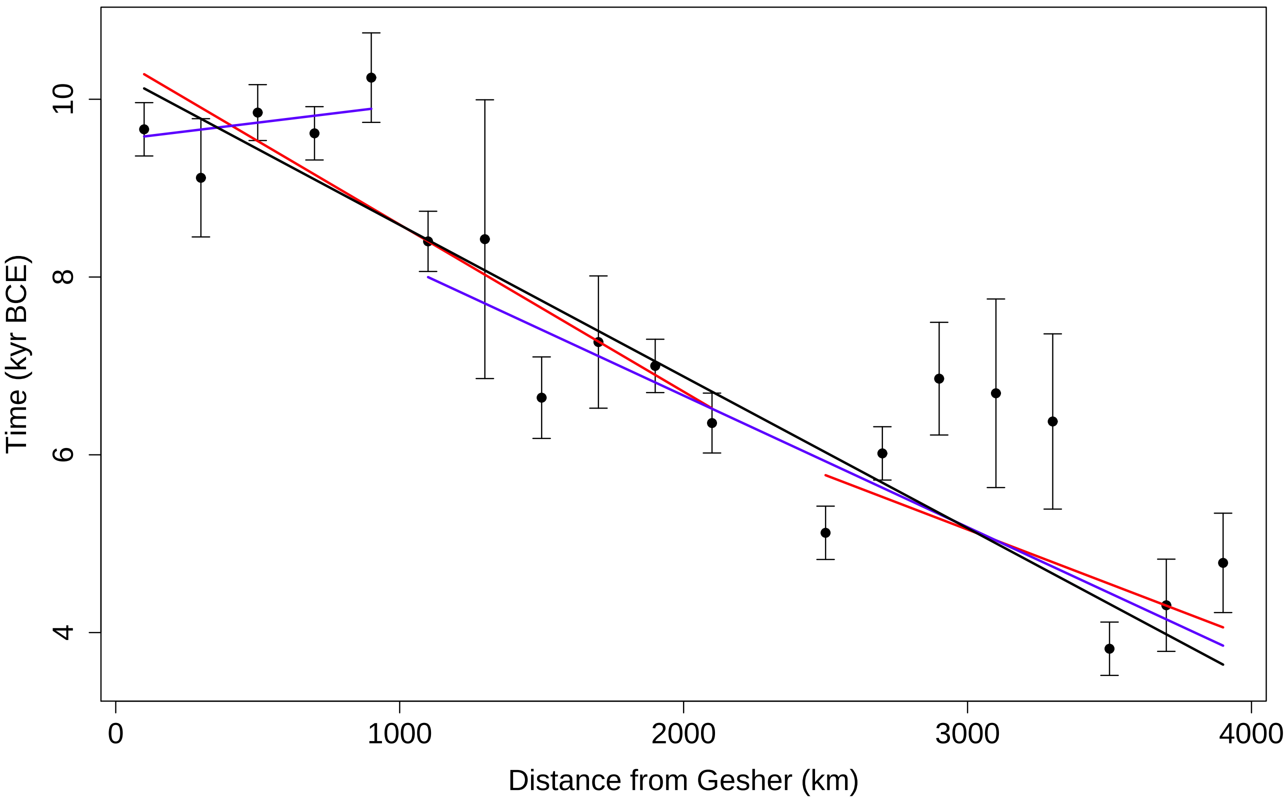

Figure S6: The best-fitting dependencies of the earliest Neolithic age T_0 at a distance D from Gesher: the constant-speed fit (black), the continuous variable-speed fit (red) and the discontinuous variable-speed fit (blue). The data fitted are the 95%-ile points of the binned data.

using the maximum likelihood formulism lead to the same conclusions as for the weighted least squares fits; the simple constant-speed fit is the best model.

We note for completeness that, within the maximum likelihood formalism, we also carried out variants of the variable-speed fits, with different variances allowed on either side of D_0 (i.e., we then had parameters σ_1, σ_2, rather than the global parameter σ). The results for the variable-speed fits were not significantly different, and the constant-speed fit was still preferred.

References

[1] Possehl GL (1999) *Indus Age: The Beginnings* (Univ Pennsylvania Press, Philadelphia).

[2] Azarnoush M, Helwing B (2005) Recent archaeological research in Iran: Prehistory to Iron Age. *Archaeologische Mitteilungen aus Iran und Turan* 37:189–246.

[3] Böhner U, Schyle D, *Radiocarbon Context Database*, http://context-database.uni-koeln.de, accessed in 2013.

[4] Segal D, Carmi I (2003) Radiocarbon dating. *The Neolithic site of Abu Ghosh. The 1995 Excavations*, IAA Reports 19, eds Khalaily H, Marder O (Israel Antiquities Authority Publ).

[5] Platform for the publication of Neolithic radiocarbon dates. *Ex Oriente,* http://www.exoriente.org/associated_projects/ppnd.php, accessed in 2013.

[6] Rollefson GO (2005) Stone tools from Ayn Jammam, near Ras en-Naqb, Southern Jordan. *Neolithics* 1:17–23.

[7] Fazeli H, Conningham RAE, Batt CM (2004) Cheshmeh-Ali revisited: towards an absolute dating of the Late Neolithic and Chalcolithic of Iran's Tehran Plain, *Iran* 42:13–23.

[8] Riehl S et al (2011) Plant use in three Pre-Pottery Neolithic sites of the northern and eastern Fertile Crescent: a preliminary report. *Vegetation History and Archaeobotany* 21:95–106.

[9] Alizadeh A, Miller NF, Rosen AM, Redding RW (2003) *Excavations at the Prehistoric Mound of Chogha Bonut, Khuzestan, Iran. Seasons 1976/77, 1977/78, and 1996* (Oriental Institute, Univ Chicago, Ill).

[10] Dupree L et al (1972) Prehistoric research in Afghanistan (1959–1966). *Trans Amer Philos Soc* 62:1–84.

[11] Görsdorf J (2000) 14C-Datings of the Es-Sifiya Settlement (Area C). *At the Crossroads. Essays on the Archaeology, History and Current Affairs of the Middle East*, eds Bienert HD, Müller-Neuhof B, Wagner-Lux U, Liedgens I (German Protestant Institute of Archaeology in Amman), pp. 15–19

[12] Simmons AH, Najjar M (2006) Ghwair I: a Neolithic community in Southern Jordan. *J Field Archaeol* 31:77–95.

[13] CZAP : The Central Zagros Archaeological Project, Accessed in 2013 http://www.czap.org/jani

[14] Benz M, Coskun A, Weninger B, Alt K W, Ozkaya V (2010) Stratigraphy and Radiocarbon dates of the PPNA site of Körtik tepe Diyarbakir, *Arkeometri Sonuçlari Toplantisi* vol 26: 81-100

[15] Ozkaya V, Coskun A (2007) Körtik Tepe Kazilari: Erken Neolithik Dönemde Bölgesel Külturel Ilişkiler Üzerine Bazi Gözlemler, *B. Can and M. Işikli (eds.) Işik D. Arkeologji Yazilari;Atatürk Üniversitesi 50. Kuruluş Yildönümü Arkeoloji Bölümü Armağani;Istanbul* 85-98

[16] Ozkaya V, San O (2007) Körtik Tepe: Bulgular Işiğinda Külturel Doku Üzerine-Ilk Gözlemler, *Özdoğan M and Başgelen N (eds.) Anadolu'da Uygarliğin Doğuşu ve Avrupa'ya Yayilimi: Türkije'de Neolithik Dönem, Yeni Kazilar, Yeni Bulgular* 21-36

[17] CZAP : The Central Zagros Archaeological Project, Accessed in 2013 http://www.czap.org/sheikh-e-abad

[18] Alizadeh A, Miller N F, Kimiaie M, Mashkour M (2006) The Origins of state organizations in prehistoric highland FARS Southern Iran; Excavations at Tall-e Bakun; Appendix A : Tables 9;10;11, *Oriental Institute Publications* vol 128 ch 11:107-118

[19] Isakov A, Kohl P L, Lamberg-Karlovsky C C, Maddin R (1987) Metallurgical Analysis From Sarazm; Tadjikistan SSR, *Archaeometry* vol 29 num 1: 90-102

[20] Agrawal D P, Krishnamurthy R V, Kusum S (1985) Physical Research Laboratory Radiocarbon Date List V, *Archaeometry* vol 27 num 1: 95-110

[21] Lamberg-Karlovsky C C (1970) Excavations at Tepe Yahya; Iran 1967-1969 PROGRESS REPORT I, *American School of Prehistoric Research and The Asia Institute of Pahlavi University*

[22] Fujii S (2007) Wadi Abu Tulayha and Wadi Ruweishid ash-Sharqi; An investigation of PPNB barrage systems in the Jafr Basin, *Neo-Lithics* vol 2: 6-17

[23] Simmons et al (2001) Wadi Shu'eib; a large Neolithic community in central Jordan: Final report of test investigations *Bulletin of the American Schools of Oriental Research* vol 321: 1-39

[24] Gopher A (1994) Arrowheads of the Neolithlic Levant: A Seriation Analysis, *Eisenbrauns*, Dissertation series:10

[25] Hours F (1994) Atlas des sites du proche orient (14000-5700 BP), *Travaux de la Maison de l'Orient mditerranen* 24

[26] Burleigh R, Ambers J, Matthews K (1982) British Museum natural radiocarbon measurements XV, *Radiocarbon* vol 24 num 3: 262-290

[27] Rollefson G (1998) Expanded Radiocarbon Chronology from Ain Ghazal, *Neo-Lithics* vol 2: 8-10

[28] Cauvin J (1974) Les Débuts de la céramique sur le Moyen-Euphrate : nouveaux documents, *Paléorient* vol 2 num 1: 199-205

[29] Hole F, ed. Aurenche et al (1986) Chronologies in the Iranian Neolithic, Chronologies in the Near East : relative chronologies and absolute chronology 16.000-4.000 B.P. : C.N.R.S. International symposium, Lyon (France), *BAR International Series 379* series(i),24-28 Nov: 353-379

[30] Kozlowski S K (1994) Radiocarbon dates from aceramic Iraq, in Late Quaternary Chronology and paleoclimates of the eastern mediterranean ed. Bar-Yosuf O,Kra R S, *Tucson: Radiocarbon* 255-264

[31] Kozlowski S K (1989) Nemrik 9; a PPN Neolithic site in Northern Iraq, *Paléorient* vol 15 num 1:25-31

[32] Hedges et al (1996) Radiocarbon Dates from the Oxford AMS system: Archaeometry datelist 21, *Archaeometry* vol 38 num 1:181-207

[33] Watkins T, Betts A, Dobney K, Nesbitt M (1995) Qermez Dere; Tel Afar: Interim Report 3, In: Project Paper No. 14 *Department of Archaeology; University of Edinburgh*

[34] Scott EM, Cook GT, Naysmith P (2007) Error and uncertainty in radiocarbon measurements. *Radiocarbon* 49:427–440.

[35] Dolukhanov P, Shukurov A, Gronenborn D, Sokoloff D, Timofeev V, Zaitseva G (2005) The chronology of Neolithic dispersal in Central and Eastern Europe. *J Archaeol Sci* 32:1441–1458.

[36] Aitken M (1990) *Science-based Dating in Archaeology,* p. 98 (Longman, London).

[37] Mazurkevich A, Dolukhanov P, Shukurov A, Zaitseva G (2009) Late Stone – early Bronze age cites in the Western Dvina–Lovat Area. *The East European Plain on the Eve of Agriculture,* British Archaeological Reports International Series 1964, eds Dolukhanov PM, Sarson GR, Shukurov AM (Archaeopress, Oxford), pp 145–153.

[38] Dolukhanov P, Shukurov A (2004) Modelling the Neolithic dispersal in Northern Eurasia. *Documenta Praehistorica* 31:35–47.

[39] Dolukhanov P, Sokoloff D, Shukurov A (2001) Radiocarbon chronology of Upper Palaeolithic sites in Eastern Europe at improved resolution. *J Archaeol Sci* 28:699–712.

[40] Bronk Ramsey C (2009) Bayesian analysis of radiocarbon dates. *Radiocarbon* 51:337–360.

[41] Buck CE, Litton CD, Smith AFM (1992) Calibration of radiocarbon results pertaining to related archaeological events. *J Archaeol Sci* 19:497–512.

[42] Marshall JL (2012) *Missing Links: Demic Diffusion and the Development of Agriculture on the Central Iranian Plateau* (Durham Theses, Durham University, UK. Available at *Durham E-Theses Online:* http://etheses.dur.ac.uk/3547/).

[43] Baggaley AW, Sarson GR, Shukurov A, Boys RJ, Golightly A (2012) Bayesian inference for a wavefront model of the Neolithisation of Europe. *Phys Rev E* 86:016105.

[44] Baggaley AW, Boys RJ, Golightly A, Sarson GR, Shukurov A (2012) Inference for population dynamics in the Neolithic period *Ann Appl Stat* 6:1352–1376.

[45] Draper NR, Smith H (1981) *Applied Regression Analysis* (2nd ed, Wiley Publ, NY).

[46] Leonard T, Hsu JSJ (1999) *Bayesian Methods: An Analysis for Statisticians and Interdisciplinary Researchers* (Cambridge Univ Press, Cambridge, UK).

[47] Akaike H (1978) A Bayesian analysis of the minimum AIC procedure *Ann Inst Statist Math*, A30:9–14.

[48] Burnham KP, Anderson DR (2010) *Model Selection and Multi-Model Inference: A Practical Information-Theoretic Approach,* p. 66 (2nd ed, Springer, NY).

[49] Main IG, Leonard T, Papasouliotis O, Hatton CG, Meredith PG (1999) One slope or two? Detecting statistically significant breaks of slope in geophysical data, with application to fracture scaling relationships *Geophys Res Lett* 26:2801–2804.

[50] Henry DO et. al. (2003) The Early Neolithic Site of Ayn Abu Nukhayla, Southern Jordan *BASOR* 330:1–30.

[51] Marshall JL (2012) Missing Links: Demic Diffusion and the Development of Agriculture on the Central Iranian Plateau *Durham Theses, Durham University* http://etheses.dur.ac.uk/3547/.

[52] Szymczak K, Khudzhanazarov M (2006) *Exploring the Neolithic of the Kyzyk-Kums* p. 26(Institute of Archaeology, Warsaw University).

[53] eds Potts DT, Roustaei K, Petrie CA, Weeks LR (2009) *The Mamasani Archaeologica Project Stage One, A report on first two seasons of teh ICAR- University of Sydney expedition to the Mamasani District, Fars Province, Iran* (Archaeopress, Oxford).

[54] Press WH, Teukolsky SA, Vetterling WT, Flannery BP (2007) *Numerical Recipes: The Art of Scientific Computing* Third edition (Cambridge University Press).

[55] Bernback R, Pollock S, Nashli HF (2008) Rahmatabad : Dating the aceramic Neolithic in Fars province *Neo-Lithics* 1/08:37–39.

[56] Pollard AM, Davoudi H, Mostafapour I, Valipour HR, Nashli HF (2012) A New radiocarbon chronology for the late Neolithic to Iron age in the Qazvin plain, Iran. *Intl. J. Humanities* 19(3):110–151.

[57] Alibaigi S, Khosravi S (2009) Tepeh Khaleseh: a new Neolithic and Palaeolithic site in the Abharrud basin in north-western Iran *Antiquity* 83(319).

[58] Valipour HR, Davoudi H, Sadati JH, Nashli HF (2012) Tepe Khaleseh: archaeological evaluation of a Late Neolithic site in north-western Iran. *Antiquity* 86(331).
