# Supplementary material for: The Near-Eastern Roots of the Neolithic in South Asia
Source: PLoS One. 2014 May 7;9(5):e95714. doi: 10.1371/journal.pone.0095714 (PMC4012948; doi:10.1371/journal.pone.0095714)
Supplement: Appendix S1 — Supporting tables S1–S5. (PDF) [file pone.0095714.s002.pdf]

The Near-Eastern roots of the Neolithic in South Asia

Appendix: The Radiometric and Archaeological Dates

Kavita Gangal, Graeme R. Sarson and Anvar Shukurov

School of Mathematics and Statistics, Newcastle University,
Newcastle upon Tyne, NE1 7RU, United Kingdom

Table S1: The ^{14}C dates used in the analysis, arranged in the alphabetical order according to the site name. Column 2 shows the laboratory number for those dates. For some sites, we have omitted ^{14}C dates for reasons given in Table S3

No.	Lab number	Site Name	Lat. °N	Long. °E	Age (yr cal BCE)	$\tilde{\sigma}$ (yr)	Distance from Gesher (km)	Ref.
1	BM-1823	Abadah	33.97	44.83	4612	109	877	[3]
2	RT 2453	Abu Gosh	31.80	35.11	8037	213	102	[5]
3	OxA-1228	Abu Hureyra	35.87	38.40	9048	241	445	[3]
4	BM-1122	Abu Hureyra	35.87	38.40	8729	379	445	[3]
5	Pta-2699	Abu Madi I	28.56	34.00	9739	382	477	[3]
6	Pta-4568	Abu Madi I	28.56	34.00	9636	384	477	[3]
7	Pta-4552	Abu Madi I	28.56	34.00	9505	252	477	[3]
8	Pta-4577	Abu Madi I	28.56	34.00	9478	324	477	[3]
9	A-11802	Àin Abu Nukhayla	29.55	35.41	7735	206	345	[5, 50]
10	A-11806	Àin Abu Nukhayla	29.55	35.41	7726	229	345	[5, 50]
11	A-11804	Àin Abu Nukhayla	29.55	35.41	7618	97	345	[5, 50]
12	A-11803	Àin Abu Nukhayla	29.55	35.41	7480	304	345	[5, 50]
13	A-11807	Àin Abu Nukhayla	29.55	35.41	7395	199	345	[5, 50]
14	GrN-12966	Àin Ghazal	31.93	35.94	8478	237	89	[3]
15	GrN-12965	Àin Ghazal	31.93	35.94	8248	281	89	[3]
16	GrN-12960	Àin Ghazal	31.93	35.94	8207	247	89	[3]
17	GrN-12959	Àin Ghazal	31.93	35.94	8134	303	89	[3]
18	Beta-138548	Akarçay Tepe	36.92	38.02	7784	170	527	[3]
19	Beta-174035	Akarçay Tepe	36.92	38.02	7587	59	527	[3]
20	Beta-138583	Akarçay Tepe	36.92	38.02	7370	228	527	[3]
21	P-1499	Ali Agha	36.45	43.82	5844	137	869	[3]
22	SI-160	Ali Kosh	32.56	47.32	8019	274	1105	[3]
23	Sh-1174	Ali Kosh	32.56	47.32	8038	501	1105	[51]
24	Beta-118721	Ali Kosh	32.56	47.32	7893	309	1105	[51]
25	Beta-536	Aqab	37.08	40.82	5120	181	690	[3]
26	OxA-574	Arjoune	34.56	36.55	5414	191	233	[3]
27	TB-300	Arukhlo 1	41.00	43.87	6029	180	1188	[3]
28	OxA-7882*	Ashkelon	31.61	34.50	6954	340	150	[3]
29	OxA-7883*	Ashkelon	31.61	34.50	6893	248	150	[3]
30	OxA-7995*	Ashkelon	31.61	34.50	6881	180	150	[3]
31	OxA-7916*	Ashkelon	31.61	34.50	6860	179	150	[3]
32	GrN-6434	Asiab	34.30	47.19	9117	282	1097	[3]
33	GrN-6413	Asiab	34.30	47.19	9144	305	1097	[51]
34	GrN-19116	Asikli Höyük	38.35	34.23	8106	165	644	[3]
35	Hd-19640	Asikli Höyük	38.35	34.23	8036	195	644	[3]
36	GrN-19865	Asikli Höyük	38.35	34.23	8003	244	644	[3]
37	GrN-20349	Asikli Höyük	38.35	34.23	7983	227	644	[3]
38	GrN-19120	Asikli Höyük	38.35	34.23	7950	272	644	[3]
39	GrN-19858	Asikli Höyük	38.35	34.23	7903	302	644	[3]
40	GrN-19115	Asikli Höyük	38.35	34.23	7891	311	644	[3]
41	GrN-19118	Asikli Höyük	38.35	34.23	7885	277	644	[3]
42	GrN-19869	Asikli Höyük	38.35	34.23	7883	289	644	[3]
43	GrN-20353	Asikli Höyük	38.35	34.23	7882	285	644	[3]
44	GrN-19870	Asikli Höyük	38.35	34.23	7881	295	644	[3]
45	GrN-20684	Asikli Höyük	38.35	34.23	7878	289	644	[3]
46	GrN-20354	Asikli Höyük	38.35	34.23	7876	290	644	[3]
47	P-1239	Asikli Höyük	38.35	34.23	7821	350	644	[3]

Continued on next page

No.	Lab number	Site Name	Lat. °N	Long. °E	Age (yr cal BCE)	$\tilde{\sigma}$ (yr)	Distance from Gesher (km)	Ref.
48	GrN-19119	Asikli Höyük	38.35	34.23	7787	173	644	[3]
49	GrN-18617	Asikli Höyük	38.35	34.23	7773	167	644	[3]
50	GrN-18618	Asikli Höyük	38.35	34.23	7772	169	644	[3]
51	GrN-18620	Asikli Höyük	38.35	34.23	7771	172	644	[3]
52	GrN-19860	Asikli Höyük	38.35	34.23	7770	169	644	[3]
53	GrN-20352	Asikli Höyük	38.35	34.23	7770	169	644	[3]
54	GrN-19861	Asikli Höyük	38.35	34.23	7760	177	644	[3]
55	GrN-19121	Asikli Höyük	38.35	34.23	7712	223	644	[3]
56	GrN-20351	Asikli Höyük	38.35	34.23	7687	96	644	[3]
57	GrN-19360	Asikli Höyük	38.35	34.23	7677	75	644	[3]
58	GrN-19363	Asikli Höyük	38.35	34.23	7667	67	644	[3]
59	GrN-19867	Asikli Höyük	38.35	34.23	7665	90	644	[3]
60	GrN-19362	Asikli Höyük	38.35	34.23	7651	65	644	[3]
61	GrN-19863	Asikli Höyük	38.35	34.23	7651	61	644	[3]
62	GrN-18619	Asikli Höyük	38.35	34.23	7646	100	644	[3]
63	GrN-19361	Asikli Höyük	38.35	34.23	7640	108	644	[3]
64	GrN-19868	Asikli Höyük	38.35	34.23	7633	306	644	[3]
65	GrN-19364	Asikli Höyük	38.35	34.23	7627	89	644	[3]
66	GrN-19862	Asikli Höyük	38.35	34.23	7625	92	644	[3]
67	GrN-19359	Asikli Höyük	38.35	34.23	7623	126	644	[3]
68	GrN-20356	Asikli Höyük	38.35	34.23	7620	103	644	[3]
69	GrN-19358	Asikli Höyük	38.35	34.23	7610	123	644	[3]
70	MC-865	Assouad	36.58	39.00	7836	363	541	[3]
71	LY-11385	Aswad	33.42	36.53	9289	459	127	
72	Gif-2633	Aswad	33.42	36.53	9198	447	127	[3]
73	Gif-2372	Aswad	33.42	36.53	8979	322	127	[3]
74	Pta-3950	Atlit-Yam	32.55	34.91	6907	258	58	[3]
75	Gif/LSM-11205	Ayakagytma	40.65	64.62	6045	33	2729	[52]
76	Gif-11206 / GifA-99182	Ayakagytma	40.65	64.62	5997	226	2729	[52]
77	Gif-11207 / GifA-99183	Ayakagytma	40.65	64.62	5990	218	2729	[52]
78	Gif/LSM-11103	Ayakagytma	40.65	64.62	5987	61	2729	[52]
79	Gif/LSM-11104	Ayakagytma	40.65	64.62	5984	74	2729	[52]
80	Gif-11208 / GifA-99181	Ayakagytma	40.65	64.62	5895	161	2729	[52]
81	Gif-11101 / GifA-99148	Ayakagytma	40.65	64.62	5869	142	2729	[52]
82	Gif-11099 / GifA-99131	Ayakagytma	40.65	64.62	5862	144	2729	[52]
83	Gif/LSM-11102	Ayakagytma	40.65	64.62	5835	60	2729	[52]
84	Gif-10660	Ayakagytma	40.65	64.62	5681	166	2729	[52]
85	Gif-11105	Ayakagytma	40.65	64.62	5563	80	2729	[52]
86	OxA-870	Azraq 31	31.83	36.82	7334	251	153	[3]
87	Bln-5123	Ba´ja	30.41	35.46	7110	68	249	[3]
88	Bln-5036	Ba´ja	30.41	35.46	6840	190	249	[3]
89	Bln-5035	Ba´ja	30.41	35.46	6834	195	249	[3]
90	KIA-11579	Baaz	33.81	36.51	4569	111	158	[3]
91	Hd-22340	Bademağaci	37.40	30.48	6865	167	699	[3]
92	TF-786	Bagor	25.35	74.38	5146	425	3844	[1]
93	P-931	Bakun B	29.85	52.83	5246	216	1673	[51]
94	P-438	Bakun B	29.85	52.83	4948	257	1673	[51]
95	UCLA-1923A	Balakot	25.48	66.73	4019	318	3124	[1]

Continued on next page

No.	Lab number	Site Name	Lat. °N	Long. °E	Age (yr cal BCE)	$\tilde{\sigma}$ (yr)	Distance from Gesher (km)	Ref.
96	UCLA-1923B	Balakot	25.48	66.73	3992	221	3124	[1]
97	GrN-14537	Basta	30.23	35.53	7384	208	269	[3]
98	GrN-14538	Basta	30.23	35.53	7185	128	269	[3]
99	P-1380	Beidha	30.37	35.45	8313	321	254	[3]
100	GrN-5062	Beidha	30.37	35.45	8155	166	254	[3]
101	GrN-5063	Beidha	30.37	35.45	8009	215	254	[3]
102	P-1382	Beidha	30.37	35.45	7976	315	254	[3]
103	GrN-5136	Beidha	30.37	35.45	7964	242	254	[3]
104	K-1410	Beidha	30.37	35.45	7955	297	254	[3]
105	P-1381	Beidha	30.37	35.45	7902	304	254	[3]
106	K-1082	Beidha	30.37	35.45	7892	317	254	[3]
107	P-1379	Beidha	30.37	35.45	7644	293	254	[3]
108	P-19c	Belt	36.65	53.28	6506	970	1682	[51]
109	RT-1394	Betzet 1	33.07	35.15	7329	244	58	[3]
110	GrN-8262	Bouqras	35.03	40.39	7442	101	522	[3]
111	GrN-13080	Bouqras	35.03	40.39	7427	118	522	[3]
112	GrN-13102	Bouqras	35.03	40.39	7426	91	522	[3]
113	GrN-8263	Bouqras	35.03	40.39	7355	212	522	[3]
114	GrN-13104	Bouqras	35.03	40.39	7338	155	522	[3]
115	GrN-13103	Bouqras	35.03	40.39	7334	158	522	[3]
116	GrN-4852	Bouqras	35.03	40.39	7286	226	522	[3]
117	GrN-13101	Bouqras	35.03	40.39	7265	182	522	[3]
118	GrN-8261	Bouqras	35.03	40.39	7183	125	522	[3]
119	GrN-8258	Bouqras	35.03	40.39	7168	128	522	[3]
120	OxA-2770	Burqu 35	31.95	37.20	7289	208	176	[3]
121	OxA-2769	Burqu 35	31.95	37.20	7255	213	176	[3]
122	OxA-2768	Burqu 35	31.95	37.20	7140	314	176	[3]
123	GrN-1544	Byblos	34.12	35.65	6231	159	164	[3]
124	Ly-4437	Cafer	38.42	38.75	8061	234	705	[3]
125	Ly-2523	Cafer	38.42	38.75	7773	407	705	[3]
126	HU-11	Canhasan III	37.25	33.37	7635	115	548	[3]
127	HU-12	Canhasan III	37.25	33.37	7601	115	548	[3]
128	BM-1667R	Canhasan III	37.25	33.37	7470	281	548	[3]
129	BM-1662R	Canhasan III	37.25	33.37	7455	268	548	[3]
130	OxA-9778	Catalhöyük East	37.65	32.82	7269	187	608	[3]
131	OxA-9777	Catalhöyük East	37.65	32.82	7186	128	608	[3]
132	OxA-9893	Catalhöyük East	37.65	32.82	7185	128	608	[3]
133	OxA-9892	Catalhöyük East	37.65	32.82	7183	128	608	[3]
134	P-779	Catalhöyük East	37.65	32.82	7178	336	608	[3]
135	OxA-9775	Catalhöyük East	37.65	32.82	7063	236	608	[3]
136	OxA-9948	Catalhöyük East	37.65	32.82	7062	234	608	[3]
137	AA-47057	Catalhöyük East	37.65	32.82	7043	265	608	[3]
138	P-782	Catalhöyük East	37.65	32.82	7020	323	608	[3]
139	PL-972425A	Catalhöyük East	37.65	32.82	7003	303	608	[3]
140	AA-18104	Catalhöyük East	37.65	32.82	6998	181	608	[3]
141	P-1370	Catalhöyük East	37.65	32.82	6977	324	608	[3]
142	P-775	Catalhöyük East	37.65	32.82	6976	319	608	[3]
143	OxA-9949	Catalhöyük East	37.65	32.82	6959	183	608	[3]
144	PL-9800565A	Catalhöyük East	37.65	32.82	6939	243	608	[3]
145	GrN-8103	Cayönü	38.22	39.73	10368	250	727	[3]
146	UCLA-305	Chagha Sefid	32.63	47.26	10252	428	1099	[3]
147	OxA-9994	Cheshmeh Ali	27.31	61.41	5144	147	2556	[7]
148	OxA-9995	Cheshmeh Ali	27.31	61.41	5109	109	2556	[7]

Continued on next page

No.	Lab number	Site Name	Lat. °N	Long. °E	Age (yr cal BCE)	$\tilde{\sigma}$ (yr)	Distance from Gesher (km)	Ref.
149	OxA-9996	Cheshmeh Ali	27.31	61.41	5092	129	2556	[7]
150	OxA-9855	Cheshmeh Ali	27.31	61.41	5010	202	2556	[7]
151	OxA-9956	Cheshmeh Ali	27.31	61.41	4841	116	2556	[7]
152	OxA-9937	Cheshmeh Ali	27.31	61.41	4834	129	2556	[7]
153	Erl-14835	Chia Sabz	33.34	47.14	8436	124	1086	[8]
154	Erl-14836	Chia Sabz	33.34	47.14	8107	141	1086	[8]
155	KIA43836	Čoḡā Golān	33.38	46.27	8694	123	1005	[8]
156	Beta-104553	Čoḡā Bonut	32.22	48.51	10125	446	1219	[9]
157	Beta-106167	Čoḡā Mīš	32.21	48.55	7348	172	1223	[9]
158	UtC-1094/-1095/-1096	Damishliyah	36.49	39.03	6794	287	534	[3]
159	UtC-1124	Damishliyah	36.49	39.03	6574	181	534	[3]
160	UtC-1097/-1098/-1099	Damishliyah	36.49	39.03	6533	100	534	[3]
161	AA-38142	Dhra	31.27	35.58	9688	332	154	[3]
162	AA-38141	Dhra	31.27	35.58	9659	345	154	[3]
163	ISGS-2898	Dhra	31.27	35.58	9630	378	154	[3]
164	AA-38144	Dhra	31.27	35.58	9557	257	154	[3]
165	AA-38143	Dhra	31.27	35.58	9550	252	154	[3]
166	ISGS-A0246	Dhra	31.27	35.58	9462	196	154	[3]
167	ISGS-A0248	Dhra	31.27	35.58	9413	232	154	[3]
168	OxA-1637	Dhuweila	32.03	37.25	7362	220	177	[3]
169	BM-2349	Dhuweila	32.03	37.25	7253	195	177	[3]
170	Ly-12110	Djade	36.65	38.19	8975	202	507	[3]
171	Ly-11329	Djade	36.65	38.19	8877	244	507	[3]
172	Ly-11330	Djade	36.65	38.19	8727	380	507	[3]
173	Ly-8842	Djade	36.65	38.19	8686	125	507	[3]
174	Ly-4400	El Kowm 2	35.22	38.83	6932	249	419	[3]
175	Bln 4973	Es Sifiya	31.44	35.82	6951	175	137	[11]
176	Bln 4969	Es Sifiya	31.44	35.82	6925	149	137	[11]
177	Bln 4971	Es Sifiya	31.44	35.82	6886	176	137	[11]
178	Bln 4972	Es Sifiya	31.44	35.82	6881	178	137	[11]
179	Bln 4968	Es Sifiya	31.44	35.82	6876	176	137	[11]
180	ISGS-3279	Es Sifiya	31.44	35.82	6848	195	137	[11]
181	Bln 4970	Es Sifiya	31.44	35.82	6843	188	137	[11]
182	HD-12335	Feinan	30.62	35.43	5348	122	226	[3]
183	HD-12338	Feinan	30.62	35.43	5062	225	226	[3]
184	OxA-2100	Ganj Dareh	34.42	47.57	8164	370	1133	[3]
185	B-108245	Ganj Dareh	34.42	47.57	8116	154	1133	[3]
186	B-108242	Ganj Dareh	34.42	47.57	8116	154	1133	[3]
187	B-108243	Ganj Dareh	34.42	47.57	8106	165	1133	[3]
188	SI-4741	Ganj Dareh	34.42	47.57	8068	224	1133	[3]
189	B-108239	Ganj Dareh	34.42	47.57	8063	217	1133	[3]
190	P-1484	Ganj Dareh	34.42	47.57	8048	296	1133	[3]
191	B-108248	Ganj Dareh	34.42	47.57	8045	204	1133	[3]
192	B-108246	Ganj Dareh	34.42	47.57	8024	207	1133	[3]
193	P-1486	Ganj Dareh	34.42	47.57	8004	279	1133	[3]
194	B-108244	Ganj Dareh	34.42	47.57	7983	227	1133	[3]
195	B-108249	Ganj Dareh	34.42	47.57	7983	227	1133	[3]
196	B-108247	Ganj Dareh	34.42	47.57	7979	230	1133	[3]
197	OxA-2101	Ganj Dareh	34.42	47.57	7955	297	1133	[3]
198	OxA-2099	Ganj Dareh	34.42	47.57	7932	318	1133	[3]
199	B-108238	Ganj Dareh	34.42	47.57	7896	284	1133	[3]

Continued on next page

No.	Lab number	Site Name	Lat. °N	Long. °E	Age (yr cal BCE)	$\tilde{\sigma}$ (yr)	Distance from Gesher (km)	Ref.
200	B-108240	Ganj Dareh	34.42	47.57	7896	284	1133	[3]
201	OxA-2102	Ganj Dareh	34.42	47.57	7875	327	1133	[3]
202	P-1494	Gawra	36.43	43.34	5874	146	830	[3]
203	RT-814a	Gesher	32.65	35.52	10459	348	0	[3]
204	ISGS 4366	Ghuwayr 1	30.62	35.53	9151	506	226	[12]
205	RT-777A	Gilgal	32.00	35.44	9628	477	69	[3]
206	RT-777B	Gilgal	32.00	35.44	9585	836	69	[3]
207	Pta-4588	Gilgal	32.00	35.44	9502	244	69	[3]
208	Pta-4583	Gilgal	32.00	35.44	9309	348	69	[3]
209	Hd-20036	Göbekli Tepe	37.22	38.92	8967	208	595	[3]
210	Hd-20025	Göbekli Tepe	37.22	38.92	8845	286	595	[3]
211	Beta-13216	Gritille	37.55	38.57	7779	427	611	[3]
212	AA-41602	Hacilar	37.57	30.07	6334	96	738	[3]
213	AA-41603	Hacilar	37.57	30.07	6328	96	738	[3]
214	AA-41604	Hacilar	37.57	30.07	6256	159	738	[3]
215	P-313A	Hacilar	37.57	30.07	6228	174	738	[3]
216	P-314	Hacilar	37.57	30.07	6216	185	738	[3]
217	P-313	Hacilar	37.57	30.07	6022	209	738	[3]
218	P-455	Hajji Firuz	37.04	45.54	6175	185	1036	[3]
219	Beta-58928	Halula	36.42	38.19	7766	173	485	[3]
220	Beta-50856	Halula	36.42	38.19	7758	187	485	[3]
221	GifA-91139	Hatoula	31.82	34.98	9903	528	105	[3]
222	GifA-91360	Hatoula	31.82	34.98	9689	428	105	[3]
223	GrN-12510	Hayaz Höyük	37.48	38.33	7348	172	595	[3]
224	Pta-3625	Hemar	31.17	35.18	7965	285	168	[3]
225	OxA-1016	Hemar	31.17	35.18	7920	312	168	[3]
226	OxA-1014	Hemar	31.17	35.18	7773	407	168	[3]
227	Bln-4609	Hoca Çeşme	40.70	26.09	6510	81	1227	[3]
228	Hd-16725/119145	Hoca Çeşme	40.70	26.09	6347	115	1227	[3]
229	GrN-19779	Hoca Çeşme	40.70	26.09	6227	135	1227	[3]
230	Hd-16724/17186	Hoca Çeşme	40.70	26.09	6122	90	1227	[3]
231	Hd-16727/17038	Hoca Çeşme	40.70	26.09	5902	109	1227	[3]
232	Hd-16726/17084	Hoca Çeşme	40.70	26.09	5896	90	1227	[3]
233	RT-1397	Horvat Galil	32.96	35.32	8564	216	39	[3]
234	HD-14219/14007	Höyücek	37.45	30.57	6372	108	698	[3]
235	HD-14218/14002	Höyücek	37.45	30.57	6370	108	698	[3]
236	HD-14217/13822	Höyücek	37.45	30.57	6218	138	698	[3]
237	Gx-864	Iblis	30.16	56.84	4970	351	2039	[51]
238	P-925	Iblis	30.16	56.84	4737	192	2039	[51]
239	P-926	Iblis	30.16	56.84	4724	184	2039	[51]
240	GrN-19351	Ilipinar	40.46	29.30	6099	108	1030	[3]
241	GrN-22046	Ilipinar	40.46	29.30	6037	190	1030	[3]
242	GrN-19354	Ilipinar	40.46	29.30	6035	51	1030	[3]
243	GrN-17046	Ilipinar	40.46	29.30	5976	71	1030	[3]
244	GrN-19795	Ilipinar	40.46	29.30	5976	77	1030	[3]
245	GrN-15085	Ilipinar	40.46	29.30	5975	90	1030	[3]

Continued on next page

Table S1 – continued from previous page

No.	Lab number	Site Name	Lat. °N	Long. °E	Age (yr cal BCE)	$\tilde{\sigma}$ (yr)	Distance from Gesher (km)	Ref.
246	GrN-19352	Ilipinar	40.46	29.30	5950	67	1030	[3]
247	GrN-22787	Ilipinar	40.46	29.30	5950	103	1030	[3]
248	GrN-15087	Ilipinar	40.46	29.30	5938	95	1030	[3]
249	GrN-24613	Ilipinar	40.46	29.30	5931	123	1030	[3]
250	GrN-17045	Ilipinar	40.46	29.30	5916	71	1030	[3]
251	GrN-19793	Ilipinar	40.46	29.30	5903	95	1030	[3]
252	GrN-15077	Ilipinar	40.46	29.30	5895	112	1030	[3]
253	GrN-17048	Ilipinar	40.46	29.30	5895	168	1030	[3]
254	GrN-24615	Ilipinar	40.46	29.30	5892	98	1030	[3]
255	GrN-19794	Ilipinar	40.46	29.30	5886	113	1030	[3]
256	GrN-17054	Ilipinar	40.46	29.30	5885	98	1030	[3]
257	GrN-22788	Ilipinar	40.46	29.30	5880	104	1030	[3]
258	GrN-19792	Ilipinar	40.46	29.30	5880	133	1030	[3]
259	GrN-24614	Ilipinar	40.46	29.30	5877	108	1030	[3]
260	GrN-17052	Ilipinar	40.46	29.30	5876	110	1030	[3]
261	GrN-18478	Ilipinar	40.46	29.30	5868	123	1030	[3]
262	GrN-17055	Ilipinar	40.46	29.30	5866	116	1030	[3]
263	GrN-17051	Ilipinar	40.46	29.30	5858	120	1030	[3]
264	GrN-17056	Ilipinar	40.46	29.30	5854	121	1030	[3]
265	GrN-24616	Ilipinar	40.46	29.30	5850	132	1030	[3]
266	GrN-17047	Ilipinar	40.46	29.30	5828	154	1030	[3]
267	GrN-15078	Ilipinar	40.46	29.30	5827	154	1030	[3]
268	GrN-16144	Ilipinar	40.46	29.30	5814	82	1030	[3]
269	GrN-16149	Ilipinar	40.46	29.30	5808	174	1030	[3]
270	GrN-19791	Ilipinar	40.46	29.30	5785	112	1030	[3]
271	GrN-22786	Ilipinar	40.46	29.30	5780	107	1030	[3]
272	GrN-19353	Ilipinar	40.46	29.30	5777	65	1030	[3]
273	GrN-19350	Ilipinar	40.46	29.30	5777	106	1030	[3]
274	GrN-21215	Ilipinar	40.46	29.30	5762	131	1030	[3]
275	GrN-22785	Ilipinar	40.46	29.30	5738	99	1030	[3]
276	GrN-22043	Ilipinar	40.46	29.30	5734	101	1030	[3]
277	GrN-16145	Ilipinar	40.46	29.30	5720	170	1030	[3]
278	GrN-19384	Ilipinar	40.46	29.30	5690	73	1030	[3]
279	GrN-18480	Ilipinar	40.46	29.30	5679	127	1030	[3]
280	GrN-18484	Ilipinar	40.46	29.30	5671	108	1030	[3]
281	GrN-18483	Ilipinar	40.46	29.30	5661	102	1030	[3]
282	GrN-24611	Ilipinar	40.46	29.30	5652	84	1030	[3]
283	GrN-17053	Ilipinar	40.46	29.30	5644	108	1030	[3]
284	GrN-22041	Ilipinar	40.46	29.30	5637	72	1030	[3]
285	OxA-2567	Iraq ed-Dubb	32.39	35.73	9561	312	35	[3]
286	KN-I 336	Iraq el-Barud	32.73	34.98	8408	199	51	[3]
287	RT-1607	Issaron	29.90	35.03	8442	159	309	[3]
288	RT-1510	Issaron	29.90	35.03	8281	286	309	[3]
289	Beta-258649	Jani	33.95	46.78	7106	239	1056	[13]
290	GrN-6353	Jarmo	35.56	44.92	6523	120	923	[3]
291	OxA-2914	Jeitun	37.95	58.23	6182	197	2137	[3]
292	OxA-2915	Jeitun	37.95	58.23	6069	178	2137	[3]
293	OxA-2916	Jeitun	37.95	58.23	6064	177	2137	[3]
294	OxA-2913	Jeitun	37.95	58.23	6059	178	2137	[3]
295	OxA-2912	Jeitun	37.95	58.23	5990	218	2137	[3]
296	OxA-4916	Jeitun	37.95	58.23	6037	395	2137	[51]
297	OxA-4694	Jeitun	37.95	58.23	6025	181	2137	[51]
298	OxA-4915	Jeitun	37.95	58.23	5940	130	2137	[51]

Continued on next page

No.	Lab number	Site Name	Lat. °N	Long. °E	Age (yr cal BCE)	$\tilde{\sigma}$ (yr)	Distance from Gesher (km)	Ref.
299	OxA-4690	Jeitun	37.95	58.23	5893	131	2137	[51]
300	OxA-4692	Jeitun	37.95	58.23	5884	135	2137	[51]
301	OxA-4693	Jeitun	37.95	58.23	5872	131	2137	[51]
302	OxA-4914	Jeitun	37.95	58.23	5834	173	2137	[51]
303	OxA-4691	Jeitun	37.95	58.23	5756	123	2137	[51]
304	Ly-10651	Jerf el Ahmar	36.39	38.20	9523	229	483	[3]
305	Ly-10648	Jerf el Ahmar	36.39	38.20	9437	218	483	[3]
306	Beta-71870	Jerf el Ahmar	36.39	38.20	9306	130	483	[3]
307	Ly-275	Jerf el Ahmar	36.39	38.20	9244	398	483	[3]
308	P-378	Jericho	31.86	35.47	9231	422	88	[3]
309	P-379	Jericho	31.86	35.47	9035	233	88	[3]
310	P-377	Jericho	31.86	35.47	8988	258	88	[3]
311	BM-1327	Jericho	31.86	35.47	8964	228	88	[3]
312	OxA-2969	Jilat 26	31.50	36.42	7898	309	153	[3]
313	OxA-2407	Jilat 26	31.50	36.42	7893	309	153	[3]
314	OxA-1802	Jilat 26	31.50	36.42	7875	327	153	[3]
315	OxA-526	Jilat 7	31.52	36.42	7919	308	152	[3]
316	OxA-527	Jilat 7	31.52	36.42	7624	313	152	[3]
317	GifA-100396	Kaletepe	38.28	34.57	8207	247	632	[3]
318	GifA-100631	Kaletepe	38.28	34.57	8134	303	632	[3]
319	GifA-99090	Kaletepe	38.28	34.57	7965	285	632	[3]
320	TF-439	Kalibangan	29.42	74.08	5412	188	3672	[1]
321	HD-10818/10747	Karain B	37.07	30.55	6267	167	669	[3]
322	Hd-10817/10764	Karain B	37.07	30.55	5949	148	669	[3]
323	TK-859	Kashkashok II	36.71	40.59	6781	275	647	[3]
324	TK-803	Kashkashok II	36.71	40.59	6619	199	647	[3]
325	GrN-26147	Khirbet Hammam	31.02	35.65	7437	94	182	[3]
326	P-524	Kili Ghul Mohammad I	30.28	66.97	4277	188	2983	[1]
327	L-180A	Kili Ghul Mohammad I	30.28	66.97	4271	1165	2983	[1]
328	UW-61	Kili Ghul Mohammad I	30.28	66.97	4074	246	2983	[1]
329	ETH-39511	Körtik Tepe	37.83	40.97	9739	292	759	[14]
330	ETH-38851	Körtik Tepe	37.83	40.97	9726	281	759	[14]
331	ETH-38849	Körtik Tepe	37.83	40.97	9639	234	759	[14]
332	ETH-38855	Körtik Tepe	37.83	40.97	9600	209	759	[14]
333	ETH-38850	Körtik Tepe	37.83	40.97	9597	206	759	[14]
334	ETH-38853	Körtik Tepe	37.83	40.97	9568	230	759	[14]
335	ETH-38854	Körtik Tepe	37.83	40.97	9540	213	759	[14]
336	ETH-38848	Körtik Tepe	37.83	40.97	9529	212	759	[14]
337	ETH-39509	Körtik Tepe	37.83	40.97	9521	231	759	[14]
338	ETH-38852	Körtik Tepe	37.83	40.97	9483	182	759	[14]
339	ETH-39512	Körtik Tepe	37.83	40.97	9479	182	759	[14]
340	ETH-39510	Körtik Tepe	37.83	40.97	9468	186	759	[14]
341	Beta-178242	Körtik Tepe	37.83	40.97	9347	89	759	[15, 16]
342	GrN-12652	Kumartepe	37.48	38.48	6849	207	601	[3]
343	HD-12915/12673	Kuruçay Höyük	37.62	30.15	6196	165	737	[3]
344	HD-12916/12674	Kuruçay Höyük	37.62	30.15	5997	71	737	[3]
345	HD-12917/12830	Kuruçay Höyük	37.62	30.15	5902	174	737	[3]
346	IGAN-772	Magzaliyah	36.39	42.33	6888	181	749	[3]

Continued on next page

Table S1 – continued from previous page

No.	Lab number	Site Name	Lat. °N	Long. °E	Age (yr cal BCE)	$\tilde{\sigma}$ (yr)	Distance from Gesher (km)	Ref.
347	Pta-3652	Megadim	32.72	34.97	5921	140	52	[3]
348	BETA-1721	Mehrgarh	29.42	67.58	8520	430	3064	[1]
349	GrN-25819	Menteşe	40.27	29.52	6368	112	1002	[3]
350	GrN-25821	Menteşe	40.27	29.52	6321	75	1002	[3]
351	GrN-25822	Menteşe	40.27	29.52	6154	83	1002	[3]
352	GrN-25823	Menteşe	40.27	29.52	6138	75	1002	[3]
353	GrN-25824	Menteşe	40.27	29.52	6117	96	1002	[3]
354	GrN-24463	Menteşe	40.27	29.52	6102	116	1002	[3]
355	GrN-24461	Menteşe	40.27	29.52	6066	147	1002	[3]
356	B-2737	Mezad Mazal	30.93	35.32	7481	125	192	[3]
357	Hv-9108	Mezad Mazal	30.93	35.32	7458	143	192	[3]
358	KN-2444	Mezad Mazal	30.93	35.32	7376	192	192	[3]
359	Hv-9107	Mezad Mazal	30.93	35.32	7346	201	192	[3]
360	Hv-9106	Mezad Mazal	30.93	35.32	7280	219	192	[3]
361	AA-49102	Mezraa Teleilat	36.98	38.00	8549	200	532	[3]
362	Gd-6150	M´lefaat	36.31	43.54	10888	258	840	[3]
363	TF-1129	Mundigak	31.58	65.50	3744	226	2817	[1]
364	Ly-4927	Munhata	32.60	35.55	6216	157	6	[3]
365	Lv-607	Mureybet	36.08	38.10	10459	348	449	[3]
366	TK-34	Mushki	29.78	52.90	7845	354	1681	[3]
367	OxA-375	Naja	31.77	36.92	6268	187	164	[3]
368	Gd-2970	Nemrik 9	36.73	42.88	9699	399	811	[3]
369	Gd-4209	Nemrik 9	36.73	42.88	9689	407	811	[3]
370	Gd-5257	Nemrik 9	36.73	42.88	9653	353	811	[3]
371	Gd-5595	Nemrik 9	36.73	42.88	9561	312	811	[3]
372	Pta-4557	Netiv HaGdud	31.98	35.38	9240	405	76	[3]
373	Pta-4555	Netiv HaGdud	31.98	35.38	9105	282	76	[3]
374	Pta-4590	Netiv HaGdud	31.98	35.38	9061	235	76	[3]
375	Pta-4556	Netiv HaGdud	31.98	35.38	9042	219	76	[3]
376	OxA-8303	Nevali Çori	37.58	38.65	8509	188	618	[3]
377	KIA-14756	Nevali Çori	37.58	38.65	8482	136	618	[3]
378	Hd-16782-351	Nevali Çori	37.58	38.65	8460	151	618	[3]
379	Hd-16783-769	Nevali Çori	37.58	38.65	8453	166	618	[3]
380	OxA-8302	Nevali Çori	37.58	38.65	8428	134	618	[3]
381	KIA-14762	Nevali Çori	37.58	38.65	8424	124	618	[3]
382	OxA-8235	Nevali Çori	37.58	38.65	8418	133	618	[3]
383	KIA-14760	Nevali Çori	37.58	38.65	8344	102	618	[3]
384	Hv-8509	Nizzanim	31.72	34.58	5710	170	136	[3]
385	OxA-5500	Pinarbasi Site A	37.48	33.03	8516	210	583	[3]
386	OxA-5501	Pinarbasi Site A	37.48	33.03	8401	165	583	[3]
387	OxA-5499	Pinarbasi Site A	37.48	33.03	8248	281	583	[3]
388	OxA-5504	Pinarbasi Site B	37.49	33.04	6283	170	584	[3]
389	OxA-5503	Pinarbasi Site B	37.49	33.04	6050	162	584	[3]
390	SMU-662	Qadesh Barnea 3	30.62	34.40	6361	237	249	[3]
391	Pta-3662	Qadesh Barnea 3	30.62	34.40	6228	168	249	[3]
392	RT-1544	Qanah	32.75	35.33	5908	150	21	[3]
393	Pta-2968	Qatif Y-3	31.41	34.32	4990	219	178	[3]
394	OxA-3752	Qermez Dere	36.38	42.45	9775	375	758	[3]
395	OxA-3756	Qermez Dere	36.38	42.45	9743	371	758	[3]
396	GrN-14539	Rahub	32.62	35.93	6292	192	39	[3]
397	GrN-4426	Ramad	33.42	36.10	7259	188	101	[3]
398	GrN-4428	Ramad	33.42	36.10	7256	203	101	[3]
399	GrN-4821	Ramad	33.42	36.10	7062	234	101	[3]

Continued on next page

No.	Lab number	Site Name	Lat. °N	Long. °E	Age (yr cal BCE)	$\tilde{\sigma}$ (yr)	Distance from Gesher (km)	Ref.
400	P-2148	Rana Ghundai	30.40	68.75	4433	100	3147	[1]
401	P-460	Ras Shamra	35.58	35.73	7365	222	326	[3]
402	P-459	Ras Shamra	35.58	35.73	7144	326	326	[3]
403	Pta-3137	Reu´el	30.10	35.10	7878	289	286	[3]
404	Pta-3202	Reu´el	30.10	35.10	7845	318	286	[3]
405	Pta-2848	Reu´el	30.10	35.10	7705	120	286	[3]
406	GrN-21319	Sabi Abyad II	36.53	39.10	7569	87	542	[3]
407	GrN-22273	Sabi Abyad II	36.53	39.10	7253	195	542	[3]
408	Pta-3821	Samir	32.82	34.95	5735	107	57	[3]
409	Pta-3820	Samir	32.82	34.95	5733	163	57	[3]
410	P-466	Sarab	34.38	47.09	6863	264	1089	[3]
411	Beta-159550	Sarab	34.38	47.09	7032	260	1089	[51]
412	Beta-159548	Sarab	34.38	47.09	6865	183	1089	[51]
413	LE2172	Sarazm	39.52	67.57	3837	128	2962	[19]
414	LE2174	Sarazm	39.52	67.57	3716	63	2962	[19]
415	LE2173	Sarazm	39.52	67.57	3673	37	2962	[19]
416	P-855	Sawwan I	34.12	43.93	6286	173	798	[3]
417	P-856	Sawwan I	34.12	43.93	6193	176	798	[3]
418	SI-2668	Seh Gabi	34.58	48.00	5163	199	1175	[51]
419	SI-2669	Seh Gabi	34.58	48.00	5110	262	1175	[51]
420	SI-2670	Seh Gabi	34.58	48.00	5000	213	1175	[51]
421	OxA-7919*	Sha´ar Hagolan	32.68	35.61	6345	98	9	[3]
422	OxA-7918*	Sha´ar Hagolan	32.68	35.61	6333	95	9	[3]
423	OxA-7917*	Sha´ar Hagolan	32.68	35.61	6263	154	9	[3]
424	OxA-7885*	Sha´ar Hagolan	32.68	35.61	6175	180	9	[3]
425	OxA-7920*	Sha´ar Hagolan	32.68	35.61	6122	100	9	[3]
426	Beta-258647	Sheikh-e Abad	34.61	47.27	9306	130	1109	[17]
427	Wk-15160	Shkarat Msaied	30.45	35.44	8405	135	245	[3]
428	Wk-15159	Shkarat Msaied	30.45	35.44	8128	164	245	[3]
429	LE-631	Shōmu	41.16	45.38	6352	118	1288	[3]
430	Bta-35081	Shu´eib	31.95	35.69	7719	245	79	[3]
431	QU-1035	Siahbid	34.50	47.25	4748	290	1105	[51]
432	P-442	Siahbid	34.50	47.25	4695	202	1105	[51]
433	GrN-9833	Sinn	35.28	40.36	7685	104	534	[3]
434	Hv 1355	Snake Cave (Ghar-i-Mar, Aq Kupruk I)	36.08	66.83	9954	657	2887	[1]
435	IGAN-769	Sotto	36.28	42.37	6335	103	746	[3]
436	IGAN-774	Sotto	36.28	42.37	6054	149	746	[3]
437	P-1391	Suberde	37.35	31.93	7281	215	616	[3]
438	P-1388	Suberde	37.35	31.93	7252	214	616	[3]
439	TO-1407	Tabaqat al-Buma	32.53	35.72	6734	268	23	[3]
440	AA-56411	Tal-e Jari B	29.85	52.96	6163	172	1685	[51]
441	AA-56410	Tal-e Jari B	29.85	52.96	6065	154	1685	[51]
442	AA-56415	Tal-e Jari B	29.85	52.96	6026	181	1685	[51]
443	AA-56412	Tal-e Jari B	29.85	52.96	5858	133	1685	[51]
444	AA65264	Tal-e Jari B	29.85	52.96	6150	88	1685	[18]
445	Beta-207565	Tal-e Jari B	29.85	52.96	5998	78	1685	[18]
446	AA63491	Tall-e Bakun A	29.91	52.89	4462	123	1676	[18]
447	Beta-210983	Tall-e Bakun A	29.91	52.89	4415	73	1676	[18]
448	Beta-207562	Tall-e Bakun A	29.91	52.89	4400	62	1676	[18]
449	AA63489	Tall-e Bakun B	29.85	52.83	5179	178	1673	[18]
450	Beta-210985	Tall-e Bakun B	29.85	52.83	5109	109	1673	[18]
451	AA63492	Tall-e Jari A	29.86	52.96	5259	207	1684	[18]

Continued on next page

Table S1 – continued from previous page

No.	Lab number	Site Name	Lat. °N	Long. °E	Age (yr cal BCE)	$\tilde{\sigma}$ (yr)	Distance from Gesher (km)	Ref.
452	Beta-207564	Tall-e Jari A	29.86	52.96	5110	111	1684	[18]
453	Beta-210982	Tall-e Jari A	29.86	52.96	4898	102	1684	[18]
454	NUTA2-12459	Tang-e Bolaghi	30.15	53.13	9939	177	1692	[51]
455	Pta-2700	Tbeik	28.82	33.94	10236	361	452	[3]
456	OxA-7886*	Tel Ali	32.70	35.56	6872	191	7	[3]
457	OxA-7921*	Tel Ali	32.70	35.56	6863	178	7	[3]
458	OxA-17739	Tepe Chahar Boneh	35.80	50.03	5751	85	1378	[56]
459	OxA-17740	Tepe Chahar Boneh	35.80	50.03	5805	76	1378	[56]
460	OxA-17741	Tepe Chahar Boneh	35.80	50.03	5800	76	1378	[56]
461	OxA-17742	Tepe Chahar Boneh	35.80	50.03	5991	73	1378	[56]
462	OxA-17743	Tepe Chahar Boneh	35.80	50.03	5921	77	1378	[56]
463	OxA-17744	Tepe Chahar Boneh	35.80	50.03	5717	74	1378	[56]
464	OxA-17704	Tepe Chahar Boneh	35.80	50.03	5176	120	1378	[56]
465	OxA-17745	Tepe Chahar Boneh	35.80	50.03	5344	120	1378	[56]
466	OxA-17746	Tepe Chahar Boneh	35.80	50.03	5190	118	1378	[56]
467	OxA-17747	Tepe Chahar Boneh	35.80	50.03	5201	121	1378	[56]
468	OxA-17748	Tepe Chahar Boneh	35.80	50.03	5290	72	1378	[56]
469	OxA-17749	Tepe Chahar Boneh	35.80	50.03	5287	70	1378	[56]
470	OxA-17750	Tepe Chahar Boneh	35.80	50.03	5348	120	1378	[56]
471	OxA-17751	Tepe Chahar Boneh	35.80	50.03	5116	105	1378	[56]
472	OxA-17752	Tepe Chahar Boneh	35.80	50.03	5281	70	1378	[56]
473	OxA-17585	Tepe Ebrahim Abad	36.12	53.05	5144	148	1652	[56]
474	OxA-17597	Tepe Ebrahim Abad	36.12	53.05	5385	84	1652	[56]
475	OxA-17598	Tepe Ebrahim Abad	36.12	53.05	5286	69	1652	[56]
476	OxA-17599	Tepe Ebrahim Abad	36.12	53.05	5104	106	1652	[56]
477	OxA-17600	Tepe Ebrahim Abad	36.12	53.05	5021	173	1652	[56]
478	OxA-17601	Tepe Ebrahim Abad	36.12	53.05	5181	119	1652	[56]
479	OxA-17602	Tepe Ebrahim Abad	36.12	53.05	5200	120	1652	[56]
480	OxA-17603	Tepe Ebrahim Abad	36.12	53.05	5446	73	1652	[56]
481	OxA-17604	Tepe Ebrahim Abad	36.12	53.05	5201	121	1652	[56]
482	OxA-17605	Tepe Ebrahim Abad	36.12	53.05	5269	57	1652	[56]
483	OxA-17606	Tepe Ebrahim Abad	36.12	53.05	5341	121	1652	[56]
484	OxA-17607	Tepe Ebrahim Abad	36.12	53.05	5546	67	1652	[56]
485	OxA-17736	Tepe Ebrahim Abad	36.12	53.05	5116	105	1652	[56]
486	OxA-17737	Tepe Ebrahim Abad	36.12	53.05	5161	129	1652	[56]
487	OxA-17738	Tepe Ebrahim Abad	36.12	53.05	5171	120	1652	[56]
488	PRL-749	Tepe Gaz tavila	28.34	56.58	5604	374	2070	[20]
489	PRL-744	Tepe Gaz tavila	28.34	56.58	5603	276	2070	[20]
490	PRL-748	Tepe Gaz tavila	28.34	56.58	5599	372	2070	[20]
491	Beta-177177	Tepe Guran	33.73	47.07	7327	143	1081	[51]
492	Beta-147122	Tepe Guran	33.73	47.07	7187	122	1081	[51]
493	Beta-177116	Tepe Guran	33.73	47.07	7173	126	1081	[51]
494	Beta-147118	Tepe Guran	33.73	47.07	7002	173	1081	[51]
495	Beta-147120	Tepe Guran	33.73	47.07	6983	158	1081	[51]
496	OxA-14739	Tepe Pardis	35.45	51.60	4768	77	1512	[51]
497	OxA-14738	Tepe Pardis	35.45	51.60	3927	116	1512	[51]
498	OxA-14737	Tepe Pardis	35.45	51.60	3862	96	1512	[51]
499	OxA-14740	Tepe Pardis	35.45	51.60	4895	98	1512	[51]
500	OxA-14741	Tepe Pardis	35.45	51.60	4809	92	1512	[51]
501	OxA-14742	Tepe Pardis	35.45	51.60	4879	105	1512	[51]
502	OxA-14743	Tepe Pardis	35.45	51.60	4856	107	1512	[51]
503	OxA-14744	Tepe Pardis	35.45	51.60	4893	97	1512	[51]
504	OxA-14745	Tepe Pardis	35.45	51.60	5060	148	1512	[51]

Continued on next page

Table S1 – continued from previous page

No.	Lab number	Site Name	Lat. °N	Long. °E	Age (yr cal BCE)	$\tilde{\sigma}$ (yr)	Distance from Gesher (km)	Ref.
505	OxA-14746	Tepe Pardis	35.45	51.60	5183	121	1512	[51]
506	OxA-14747	Tepe Pardis	35.45	51.60	5183	126	1512	[51]
507	OxA-14749	Tepe Pardis	35.45	51.60	5106	111	1512	[51]
508	OxA-14750	Tepe Pardis	35.45	51.60	5107	107	1512	[51]
509	KIA33174	Tepe Rahmatabad	30.11	53.06	6866	160	1687	[55]
510	KIA33173	Tepe Rahmatabad	30.11	53.06	6924	150	1687	[55]
511	UZ5331/ETH31882	Tepe Rahmatabad	30.11	53.06	6846	200	1687	[55]
512	OxA-22347	Tepe Sialk	33.97	51.40	5200	120	1481	[51]
513	OxA-22505	Tepe Sialk	33.97	51.40	5108	104	1481	[51]
514	OxA-22504	Tepe Sialk	33.97	51.40	5154	128	1481	[51]
515	OxA-22503	Tepe Sialk	33.97	51.40	5195	119	1481	[51]
516	OxA-22502	Tepe Sialk	33.97	51.40	5351	119	1481	[51]
517	OxA-22501	Tepe Sialk	33.97	51.40	5105	105	1481	[51]
518	OxA-22500	Tepe Sialk	33.97	51.40	5113	102	1481	[51]
519	OxA-22499	Tepe Sialk	33.97	51.40	5122	100	1481	[51]
520	OxA-22498	Tepe Sialk	33.97	51.40	5157	129	1481	[51]
521	OxA-22497	Tepe Sialk	33.97	51.40	5171	119	1481	[51]
522	OxA-22496	Tepe Sialk	33.97	51.40	5161	126	1481	[51]
523	OxA-22495	Tepe Sialk	33.97	51.40	5076	131	1481	[51]
524	OxA-22494	Tepe Sialk	33.97	51.40	4875	89	1481	[51]
525	OxA-22508	Tepe Sialk	33.97	51.40	5341	122	1481	[51]
526	OxA-22507	Tepe Sialk	33.97	51.40	5344	121	1481	[51]
527	OxA-22506	Tepe Sialk	33.97	51.40	5271	59	1481	[51]
528	PRL-749	Tepe Yahya	28.20	55.98	5604	374	2020	[51]
529	PRL-744	Tepe Yahya	28.20	55.98	5603	276	2020	[51]
530	PRL-748	Tepe Yahya	28.20	55.98	5599	372	2020	[51]
531	TK-198	Thalathat II	36.48	42.50	6744	280	768	[3]
532	Bln-719	Togolok	37.98	57.83	6206	191	2103	[3]
533	AA56351	Tol-e Basi	30.08	52.59	6146	85	1644	[51]
534	AA56355	Tol-e Basi	30.08	52.59	6009	88	1644	[51]
535	AA56354	Tol-e Basi	30.08	52.59	5995	78	1644	[51]
536	AA-56340	Tol-e Basi	30.08	52.59	5986	87	1644	[51]
537	AA-56353	Tol-e Basi	30.08	52.59	5959	73	1644	[51]
538	AA-56343	Tol-e Basi	30.08	52.59	5864	117	1644	[51]
539	AA56339	Tol-e Basi	30.08	52.59	5854	122	1644	[51]
540	WK13990	Tol-e Nurabad	30.12	51.52	5863	122	1543	[53]
541	WK13991	Tol-e Nurabad	30.12	51.52	5854	128	1543	[53]
542	OZI128	Tol-e Nurabad	30.12	51.52	5854	123	1543	[53]
543	WK13992	Tol-e Nurabad	30.12	51.52	5850	127	1543	[53]
544	WK13993	Tol-e Nurabad	30.12	51.52	5728	106	1543	[53]
545	Pta-2703	Ujrat el-Mehed 1	28.58	33.93	7265	202	477	[3]
546	NITA2-11408	Wadi Abu Tulayha	30.51	35.97	7525	67	242	[5, 22]
547	NITA2-11409	Wadi Abu Tulayha	30.51	35.97	7478	109	242	[5, 22]
548	NITA2-11406	Wadi Abu Tulayha	30.51	35.97	7466	108	242	[5, 22]
549	Beta-120210	Wadi Faynan 16	30.62	35.50	9973	281	226	[3]
550	Beta-135111	Wadi Faynan 16	30.62	35.50	9973	281	226	[3]
551	Beta-35080/WS-1	Wadi Shu´eib	31.97	35.73	9961	675	78	[5, 23]
552	Pta 2700	Wadi Tbeik	28.77	33.95	10236	361	457	[5]
553	P-1244	Yanik	37.99	45.95	5889	137	1116	[3]
554	P-1243	Yanik	37.99	45.95	5827	156	1116	[3]
555	LE-1086	Yarim Tepe I	36.31	42.43	6034	188	753	[3]
556	Pta-4242	Yiftahel	32.72	35.18	7999	274	33	[3]

Continued on next page

Table S1 – continued from previous page

**No.**	**Lab number**	**Site Name**	**Lat. °N**	**Long. °E**	**Age (yr cal BCE)**	$\tilde{\sigma}$ **(yr)**	**Distance from Gesher (km)**	**Ref.**
557	RT-736b	Yiftahel	32.72	35.18	7973	318	33	[3]
558	Pta-4245	Yiftahel	32.72	35.18	7878	289	33	[3]
559	Rome-467	Yümüktepe	36.79	34.60	6832	226	468	[3]
560	Rome-734	Yümüktepe	36.79	34.60	6740	282	468	[3]
561	R-1344	Yümüktepe	36.79	34.60	6624	187	468	[3]
562	R-1343	Yümüktepe	36.79	34.60	6509	139	468	[3]
563	TUNC-12	Zagheh	35.82	49.95	6033	188	1371	[51]
564	WK 9633	ZahratAdhDhra´2(ZAD 2)	31.25	35.57	9032	210	156	[5]
565	Wk 9568	ZahratAdhDhra´2(ZAD 2)	31.25	35.57	9012	246	156	[5]
566	Wk 9447	ZahratAdhDhra´2(ZAD 2)	31.25	35.57	9011	210	156	[5]
567	Wk 9445	ZahratAdhDhra´2(ZAD 2)	31.25	35.57	8965	219	156	[5]
568	Wk 9570	ZahratAdhDhra´2(ZAD 2)	31.25	35.57	8907	250	156	[5]
569	OZE-605	ZahratAdhDhra´2(ZAD 2)	31.25	35.57	8881	244	156	[5]
570	OZE-607	ZahratAdhDhra´2(ZAD 2)	31.25	35.57	8873	247	156	[5]
571	OZE-606	ZahratAdhDhra´2(ZAD 2)	31.25	35.57	8843	272	156	[5]

Table S2: In this table we give one date per site for the first Neolithic arrival. The method of selection is mentioned in Column 2 of the table. (See the notes at the end of the table for a description of methods of selection.) SI: figure S3b also shows the various steps in this selection procedure. For the sites with archeological time periods, we use the start of the period and thus question of selection of one date does not arise there. These times are given in the column 'Age' of tables S4 and S5.

**No.**	**Method of selection**[†]	**Site Name**	**Lat. °N**	**Long. °E**	**Age (yr cal BCE)**	$\tilde{\sigma}$ **(yr)**	**Distance from Gesher (km)**
1	O	Abadah	33.97	44.83	4612	175	877
2	O	Abu Gosh	31.80	35.11	8037	213	102
3	A	Abu Hureyra	35.87	38.40	8888	226	445
4	A	Abu Madi I	28.56	34.00	9589	175	477
5	A	Àin Abu Nukhayla	29.55	35.41	7591	175	345
6	A	Àin Ghazal	31.93	35.94	8267	175	89
7	C	Akarçay Tepe	36.92	38.02	7580	228	527
8	O	Ali Agha	36.45	43.82	5844	175	869
9	A	Ali Kosh	32.56	47.32	7983	175	1105
10	O	Aqab	37.08	40.82	5120	181	690
11	A	Arjoune	34.56	36.55	5414	191	233
12	O	Arukhlo 1	41.00	43.87	6029	180	1188
13	A	Ashkelon	31.61	34.50	6897	175	150
14	A	Asiab	34.30	47.19	9130	175	1097
15	C	Asikli Höyük	38.35	34.23	7897	350	644
16	A	Assouad	36.58	39.00	7836	363	541
17	A	Aswad	33.42	36.53	9088	175	127
18	A	Atlit-Yam	32.55	34.91	6907	258	58
19	C	Ayakagytma	40.65	64.62	5882	226	2729
20	O	Azraq 31	31.83	36.82	7334	251	153
21	A	Ba´ja	30.41	35.46	6928	175	249
22	A	Baaz	33.81	36.51	4569	175	158
23	A	Bademağaci	37.40	30.48	6865	175	699
24	A	Bagor	25.35	74.38	5146	425	3844
25	C	Bakun B	29.85	52.83	5120	258	1673
26	A	Balakot	25.48	66.73	4005	175	3124
27	A	Basta	30.23	35.53	7284	175	269
28	C	Beidha	30.37	35.45	7979	321	254
29	O	Belt	36.65	53.28	6506	970	1682
30	A	Betzet 1	33.07	35.15	7329	244	58
31	C	Bouqras	35.03	40.39	7357	259	522
32	A	Burqu 27	31.95	37.20	7228	175	176
33	O	Byblos	34.12	35.65	6231	175	164
34	A	Cafer	38.42	38.75	7917	203	705
35	A	Canhasan III	37.25	33.37	7540	175	548
36	A	Catalhöyük East	37.65	32.82	7069	175	608
37	A	Cayönü	38.22	39.73	10368	250	727
38	A	Chagha Sefid	32.63	47.26	10252	428	1099
39	A	Cheshmeh Ali	27.31	61.41	5005	175	2556
40	A	Chia Sabz	33.34	47.14	8272	233	1086
41	A	Čogā Golān	33.38	46.27	8694	175	1005
42	A	Čogā Bonut	32.22	48.51	10125	446	1219
43	A	Čogā Mīš	32.21	48.55	7348	175	1223
44	A	Damishliyah	36.49	39.03	6633	175	534
45	C	Dhra	31.27	35.58	9565	378	154
46	A	Dhuweila	32.03	37.25	7307	175	177
47	A	Djade	36.65	38.19	8816	175	507
48	A	El Kowm 1	35.24	38.82	6932	249	419

Continued on next page

Table S2 – continued from previous page

No.	Method of selection†	Site Name	Lat. °N	Long. °E	Age (yr cal BCE)	$\tilde{\sigma}$ (yr)	Distance from Gesher (km)
49	A	Es Sifiya	31.44	35.82	6887	175	137
50	A	Feinan	30.62	35.43	5205	202	226
51	C	Ganj Dareh	34.42	47.57	8021	370	1133
52	A	Gawra	36.43	43.34	5874	175	830
53	A	Gesher	32.65	35.52	10458	348	0
54	A	Ghuwayr 1	30.62	35.51	9151	506	226
55	A	Gilgal	32.03	35.47	9506	175	69
56	A	Gritille	37.55	38.57	7779	427	611
57	A	Göbekli Tepe	37.22	38.92	8906	175	595
58	A	Hacilar	37.57	30.07	6230	175	738
59	A	Hajji Firuz	37.04	45.54	6175	185	1036
60	A	Halula	36.42	38.19	7762	175	485
61	A	Hatoula	31.82	34.98	9796	175	105
62	O	Hayaz Höyük	37.48	38.33	7348	175	595
63	A	Hemar	31.17	35.18	7886	175	168
64	C	Hoca Çeşme	40.70	26.09	6301	175	1227
65	A	HorseCaveAqKupruk II	36.08	66.83	9954	657	2887
66	A	Horvat Galil	32.96	35.32	8564	216	39
67	A	Höyücek	37.45	30.57	6320	175	698
68	A	Iblis	30.16	56.84	4810	175	2039
69	C	Ilipinar	40.46	29.30	5880	190	1030
70	O	Iraq ed-Dubb	32.39	35.73	9561	312	35
71	O	Iraq el-Barud	32.73	34.98	8408	199	51
72	A	Issaron	29.90	35.03	8361	175	309
73	O	Jani	33.95	46.78	7106	239	1056
74	O	Jarmo	35.56	44.92	6523	175	923
75	C	Jeitun	37.95	58.23	5969	395	2137
76	A	Jerf el Ahmar	36.39	38.20	9377	175	483
77	A	Jericho	31.86	35.47	9054	175	88
78	A	Jilat 13	31.50	36.42	7888	175	153
79	A	Jilat 7	31.52	36.42	7771	208	152
80	A	Kaletepe	38.28	34.57	8102	175	632
81	O	Kalibangan	29.42	74.08	5412	188	3672
82	A	Karain B	37.07	30.55	6108	225	669
83	A	Kashkashok II	36.71	40.59	6700	175	647
84	A	Khirbet Hammam	31.02	35.65	7437	175	182
85	A	Kili Ghul Mohammad I	30.28	66.97	4207	175	2983
86	C	Körtik Tepe	37.83	40.97	9556	292	759
87	O	Kumartepe	37.48	38.48	6849	207	601
88	A	Kuruçay Höyük	37.62	30.15	6032	175	737
89	A	M´lefaat	36.31	43.54	10888	258	840
90	A	Magzaliyah	36.39	42.33	6888	181	749
91	A	Megadim	32.72	34.97	5921	175	52
92	A	Mehrgarh	29.42	67.58	8520	430	3064
93	A	Menteşe	40.27	29.52	6181	175	1002
94	A	Mezad Mazal	30.93	35.32	7388	175	192
95	A	Mezraa Teleilat	36.98	38.00	8549	200	532
96	A	Mundigak	31.58	65.50	3744	226	2817
97	O	Munhata	32.60	35.55	6216	175	6
98	A	Mureybet	36.08	38.10	10458	348	449
99	A	Mushki	29.78	52.90	7845	354	1681
100	O	Naja	31.77	36.92	6268	187	164
101	C	Nemrik 9	36.73	42.88	9650	480	811

Continued on next page

No.	Method of selection†	Site Name	Lat. °N	Long. °E	Age (yr cal BCE)	$\tilde{\sigma}$ (yr)	Distance from Gesher (km)
102	A	Netiv HaGdud	31.98	35.38	9112	175	76
103	C	Nevali Çori	37.58	38.65	8444	311	618
104	O	Nizzanim	31.72	34.58	5710	175	136
105	C	Pinarbasi Site A	37.48	33.03	8388	281	583
106	A	Pinarbasi Site B	37.49	33.04	6166	175	584
107	A	Qadesh Barnea 3	30.62	34.40	6294	175	249
108	O	Qanah	32.75	35.33	5908	175	21
109	O	Qatif Y-3	31.41	34.32	4990	219	178
110	A	Qermez Dere	36.38	42.45	9759	175	758
111	O	Rahub	32.62	35.93	6292	192	39
112	A	Ramad	33.42	36.10	7192	175	101
113	O	Rana Ghundai	30.40	68.75	4433	175	3147
114	A	Ras Shamra	35.58	35.73	7254	175	326
115	A	Reu´el	30.10	35.10	7809	175	286
116	A	Sabi Abyad	36.53	39.10	7411	224	542
117	A	Samir	32.82	34.95	5734	175	57
118	A	Sarab	34.38	47.09	6920	175	1089
119	A	Sarazm	39.52	67.57	3742	175	2962
120	A	Sawwan I	34.12	43.93	6239	175	798
121	A	Seh Gabi	34.58	48.00	5091	175	1175
122	A	Sha´ar Hagolan	32.68	35.61	6247	175	9
123	A	Sheikh-e Abad	34.61	47.27	9306	175	1109
124	A	Shkarat Msaied	30.45	35.44	8266	196	245
125	O	Shōmu	41.16	45.38	6352	175	1288
126	O	Shu´eib	31.95	35.69	7719	245	79
127	A	Siahbid	34.50	47.25	4722	175	1105
128	A	Sinn	35.28	40.36	7685	175	534
129	A	Sotto	36.28	42.37	6194	199	746
130	A	Suberde	37.35	31.93	7267	175	616
131	A	Tabaqat al-Buma	32.53	35.72	6734	268	23
132	A	Tal-e Jari B	29.85	52.96	6043	175	1685
133	A	Tall-e Bakun A	29.91	52.89	4426	175	1676
134	C	Tall-e Jari A	29.86	52.96	5089	207	1684
135	A	Tang-e Bolaghi	30.15	53.13	9939	177	1692
136	O	Tbeik	28.82	33.94	10236	361	452
137	A	Tel Ali	32.70	35.56	6867	175	7
138	C	Tepe Chahar Boneh	35.81	50.03	6	175	1378
139	C	Tepe Ebrahim Abad	36.12	53.05	5	175	1652
140	A	Tepe Gaz Tavila	28.34	56.58	5602	175	2070
141	A	Tepe Guran	33.73	47.07	7134	175	1081
142	C	Tepe Pardis	35.45	51.60	5	175	1512
143	C	Tepe Rahmatabad	30.11	53.06	7	175	1687
144	C	Tepe Sialk	33.97	51.40	5	175	1481
145	A	Tepe Yahya	28.20	55.98	5602	175	2020
146	A	Thalathat II	36.48	42.50	6744	280	768
147	O	Togolok	37.98	57.83	6206	191	2103
148	A	Tol-e Basi	30.08	52.59	5973	175	1644
149	A	Tol-e Nurabad	30.12	51.52	5830	175	1543
150	O	Ujrat el-Mehed 1	28.58	33.93	7265	202	477
151	A	Wadi Abu Tulayha	30.51	35.97	7490	175	242
152	A	Wadi Faynan 16	30.62	35.50	9973	175	226
153	A	Wadi Shu´eib	31.97	35.73	9961	675	78
154	O	Wadi Tbeik	28.77	33.95	10236	361	457

Continued on next page

Table S2 – continued from previous page

No.	Method of selection†	Site Name	Lat. °N	Long. °E	Age (yr cal BCE)	$\tilde{\sigma}$ (yr)	Distance from Gesher (km)
155	A	Yanik	37.99	45.95	5858	175	1116
156	O	Yarim Tepe I	36.31	42.43	6034	188	753
157	A	Yiftahel	32.72	35.18	7950	175	33
158	A	Yümüktepe	36.79	34.60	6676	175	468
159	A	Zagheh	35.82	49.95	6033	188	1371
160	A	ZahratAdhDhra' 2(ZAD 2)	31.25	35.57	8941	175	156

Notes

† **C: Cluster**: The oldest Neolithic cluster at the site is used in the analysis; standard deviation of the dates in the cluster is given in σ column.
A: Average: Where the cluster method is unapplicable, we use the dates within 350 years of the oldest date. The mean of these dates within 350 years of the oldest date is given in the Age column. The associated value of σ in Column 7 is the maximum of the errors associated with each of these dates.
O: One Date: There is only a single ^{14}C date belonging to the Neolithic period at the site.

Table S3: Discarded ^{14}C dates (The reason for exclusion of the date is given in more details in the reference provided.)

No.	Lab number	Site Name	Age (yr cal BCE)	$\tilde{\sigma}$ (yr)	Reason for discarding	Ref.
3	BM-1720	Abu Hureyra	24731	826	An anomalously early date for the Neolithic, with the later dates BM-1718 and BM-1719 classified as Mesolithic. The date is also noted as suspicious.	[3, 26]
6	AA-25038	Ain Ghazal	22672	4245	An anomalously early date for the Neolithic	[27]
17	MC-607	Assouad	12662	5725	Failed attempt at ^{14}C dating (note the value of$\tilde{\sigma}$)	[28]
38	UCLA-300	Chagha Sefid	11178	214	Disturbed stratigraphy of the site, the date may have been wrongly recorded.	[3, 29]
90		M´lefaat	8970	331	Classified as unacceptable	[30]
90		M´lefaat	14392	713	Classified as unacceptable	[30]
102	Gd-5440	Nemrik 9	19888	5015	An anomalously early date for the Neolithic; classified as unacceptable	[30, 31]
102	Gd-4208	Nemrik 9	9741	450	Classified as unacceptable	[30]
102	Gd-2777	Nemrik 9	9907	528	Classified as unacceptable	[30]
102	Gd-5451	Nemrik 9	10623	349	Classified as unacceptable	[30]
102	Gd-2714	Nemrik 9	10892	259	Classified as unacceptable	[30]
102	Gd-5249	Nemrik 9	11087	243	Classified as unacceptable	[30]
111	OxA-3753	Qermez Dere	11906	255	Two samples from the same material gave divergent dates; OxA-3753 is inconsistent with the age of the site implied by other dates.	[32, 33]
118	Pta-3385	Salibiya 9	20031	421	Possibly recorded under wrong lab number.	[3]

Table S4: The archaeological dates for the Indus Valley region [1]

No.	Site Name	Lat. °N	Long. °E	Age (yr BCE)	$\tilde{\sigma}$ (yr)	Distance from Gesher (km)
1	Abduwali	28.76	71.34	3800	200	3436
2	Adhi One	28.77	71.08	3800	200	3411
3	Ahmed Khanzai North	30.18	66.97	3800	200	2986
4	Ahmed Khanzai South	30.15	66.95	3800	200	2985
5	Akkanwali Their	28.83	71.41	3800	200	3440
6	Ambrawali	28.79	71.97	3800	200	3494
7	Anjira	28.28	66.32	5000	300	2981
8	Ashal	26.06	64.42	3800	200	2885
9	Awaran Niabat	26.42	65.23	4300	300	2946
10	Azimwala Two	28.79	71.19	3800	200	3421
11	Azimwali C	28.78	71.21	3800	200	3423
12	Badalwala Five	28.69	71.08	3800	200	3414
13	Badalwala Four	28.69	71.09	3800	200	3414
14	Badrang Damb	27.67	65.52	3800	200	2927
15	Bagaya-no Timbo	23.43	71.83	4000	200	3688
16	Baggewali	28.83	71.15	3800	200	3416
17	Bahilawala B	28.85	71.48	3800	200	3446
18	Bahilawala C	28.87	71.47	3800	200	3444
19	Bajaniya-no Thumdo	23.83	71.49	4000	200	3638
20	Baleli	30.33	66.88	7000	300	2974
21	Bandwali	28.87	71.43	3800	200	3441
22	Belar Damb	27.12	66.45	4300	300	3033
23	Bhootanwala C	28.78	71.05	3800	200	3408
24	Bhootanwali Two	28.77	71.04	3800	200	3407
25	Binjor Three	29.20	73.10	4000	200	3587
26	Chak 353 West	29.19	72.27	3800	200	3509
27	Chambrawala Ther	29.33	72.30	3800	200	3508
28	Chandnewala Two	28.74	71.21	3800	200	3424
29	Changalawala C	28.85	71.38	3800	200	3437
30	Changda	22.53	72.55	4000	200	3797
31	Channanwala Ther	29.13	72.90	3800	200	3570
32	Chaudhryanwala	28.79	71.27	3800	200	3428
33	Chikrala	28.75	71.20	3800	200	3423
34	Chimri	27.82	66.63	4300	300	3025
35	Chore	28.76	71.16	3800	200	3419
36	Choteria Timbo	23.60	71.85	4000	200	3682
37	Dabar Kot	30.08	68.68	3800	200	3149
38	Dabli East	28.90	71.47	3800	200	3443
39	Dabli West	28.91	71.47	3800	200	3443
40	Damb Sadaat	30.05	66.95	3800	200	2988
41	Darkhanwala Ther	28.72	71.23	3800	200	3427
42	Datrana Eight	23.77	71.11	4000	200	3606
43	Datrana Four	23.77	71.11	3500	200	3606
44	Datrana Seven	23.78	71.12	4000	200	3606
45	Dhuni South	28.58	70.94	3800	200	3404
46	Dhuni, Hakra	28.59	70.93	3800	200	3403
47	Dosia Khal Damb	27.30	66.37	3800	200	3019
48	Drakalo Damb	27.15	66.42	3800	200	3030
49	Duki Mound	30.17	68.57	5000	300	3136
50	Faiz Mohammad	29.95	67.10	4300	300	3004
51	Gajjuwala Two	28.84	71.12	3800	200	3413
52	Ganario-no Thumdo	23.94	71.52	4000	200	3636
53	Gate Dap	26.12	64.22	3800	200	2864
54	Ghuram Damb	28.70	66.28	3800	200	2964

Continued on next page

Table S4 – continued from previous page

No.	Site Name	Lat. °N	Long. °E	Age (yr BCE)	$\tilde{\sigma}$ (yr)	Distance from Gesher (km)
55	Godavari One	22.20	69.92	4000	200	3570
56	Gokhijadio-no Timbo	23.62	71.88	4000	200	3684
57	Gudel	22.73	72.52	4000	200	3785
58	Gumla	31.88	70.83	7000	300	3306
59	Gwani Kalat	27.48	65.92	3800	200	2971
60	Harappa	30.63	72.87	3700	200	3525
61	Harhari-no Thumdo	23.88	71.39	4000	200	3627
62	Hathala	32.02	70.60	3800	200	3281
63	Hotewala Two	28.92	71.23	3800	200	3420
64	Islam Chowki	32.98	70.48	3800	200	3252
65	Isplinji One	29.69	67.05	4300	300	3007
66	Isplinji Two	29.69	67.04	5000	300	3006
67	Jafawala Three	28.71	71.14	3800	200	3418
68	Jafawala Two	28.70	71.13	3800	200	3418
69	Jalwali A	28.86	71.38	3800	200	3436
70	Jalwali B	28.86	71.38	3800	200	3436
71	Jangipar	28.68	71.08	3800	200	3414
72	Janoya-no Timbo	23.42	71.86	4000	200	3691
73	Jaren	26.22	64.75	3800	200	2909
74	Jawaiwala Two	28.73	71.07	3800	200	3411
75	Jawarji Kalat	27.52	65.87	4300	300	2965
76	Jebri Damb Two	27.29	65.75	7000	300	2962
77	Jhalar	28.71	71.12	3800	200	3417
78	Jhandewala Two	28.72	70.98	3800	200	3403
79	Kalharwala B	28.87	71.25	3800	200	3424
80	Kanewal, Sai No Tekro	22.45	72.50	4000	200	3796
81	Karezgai	30.81	67.75	3800	200	3043
82	Kargushki Damb	27.48	65.32	7000	300	2915
83	Kasiano Dozakh	30.45	66.93	7000	300	2976
84	Kechi Beg	30.12	66.95	3800	200	2986
85	Khakhar Buthi	26.32	66.27	7000	300	3047
86	KhanKandewala D	28.84	71.41	3800	200	3440
87	Khanpuri Two	28.75	71.27	3800	200	3429
88	Khiplewali	28.73	71.02	3800	200	3407
89	Khiplewali Three	28.72	71.03	3800	200	3408
90	Khiplewali Two	28.72	71.02	3800	200	3407
91	KI	29.96	66.85	3800	200	2981
92	Kikrl Two	28.72	71.33	3800	200	3436
93	Kili Ghul Mohammad	30.28	66.97	7000	300	2984
94	Killianwali	28.89	71.43	3800	200	3440
95	Killianwali D	28.88	71.45	3800	200	3442
96	Kirta	29.53	67.47	3800	200	3051
97	Kota	22.17	69.70	4000	200	3551
98	Kotada, Jamnagar	22.20	70.37	4000	200	3611
99	Kowas	30.47	67.58	3800	200	3036
100	Kuchanwala	29.11	71.91	3800	200	3478
101	Kuchnai Ghundai	30.72	67.04	3800	200	2979
102	Kuki Damb	28.75	66.35	5000	300	2969
103	Kullu Kalat	29.07	66.37	4300	300	2961
104	Kunal	29.63	75.66	3800	200	3812
105	L-2	30.30	68.17	5000	300	3095
106	L-3	30.30	68.20	5000	300	3098
107	Lak Largai	32.82	70.52	3800	200	3259
108	Lakhman	28.72	71.17	3800	200	3421
109	Lathwala Two	28.83	71.20	3800	200	3420

Continued on next page

Table S4 – continued from previous page

No.	Site Name	Lat. °N	Long. °E	Age (yr BCE)	$\tilde{\sigma}$ (yr)	Distance from Gesher (km)
110	Lewan	32.88	70.58	3800	200	3263
111	Litanwala	28.78	71.38	3800	200	3439
112	Loharki Theri	29.17	72.25	3800	200	3508
113	Loteshwar	23.60	71.84	4000	200	3681
114	Lundewali Four	28.89	71.41	3800	200	3438
115	Lundewali Three	28.89	71.41	3800	200	3438
116	Luppewala	28.82	71.21	3800	200	3422
117	Luppewala Three	28.83	71.21	3800	200	3421
118	Marki Mas	27.17	66.42	3800	200	3029
119	Mehwali Two	28.66	71.03	3800	200	3410
120	Merechi Kanda	28.82	71.24	3800	200	3424
121	Merechi Kanda Two	28.83	71.24	3800	200	3424
122	Moniwala	28.64	70.72	3800	200	3381
123	Mundigak	31.92	65.50	4300	300	2810
124	Musafarwali	28.78	71.14	3800	200	3416
125	Musafarwali Two	28.77	71.15	3800	200	3418
126	Naharnwala	28.84	71.50	3800	200	3448
127	Naharwali	28.84	71.39	3800	200	3438
128	Naharwali B	28.83	71.39	3800	200	3438
129	Nahrenwala	28.84	71.50	3800	200	3448
130	Nal	27.73	66.27	7000	300	2995
131	Nani Chandur	23.58	71.63	4000	200	3663
132	Neghar Damb	28.27	66.30	5000	300	2979
133	Niai Buthi	26.25	66.43	3800	200	3065
134	Niwaniwala Ther West	28.79	71.17	3800	200	3419
135	Niwaniwala Three	28.79	71.17	3800	200	3419
136	Nundara	26.47	65.42	3800	200	2962
137	Oinwala Ther	28.84	71.38	3800	200	3437
138	Old Balor	26.05	64.42	4300	300	2886
139	Oriyo Timbo	21.89	71.60	4000	200	3740
140	Pal	22.30	70.72	4000	200	3639
141	Panju Damb	27.32	66.42	4300	300	3023
142	Parhara	28.75	71.19	3800	200	3422
143	Parharewala B	28.07	71.18	3800	200	3443
144	Payunewala Bhit Three	28.81	71.37	3800	200	3437
145	Payunewala Bhit Two	28.98	71.37	3800	200	3432
146	Periano Ghundai	31.37	69.38	4300	300	3182
147	Phusi Damb	27.08	66.18	4300	300	3010
148	Pir Haidar Shahr	28.27	66.10	4300	300	2961
149	Q-06	29.77	66.97	4300	300	2997
150	Q-17	30.23	66.90	5000	300	2978
151	Q-18	30.18	66.88	4300	300	2978
152	Q-23	30.27	66.98	4300	300	2985
153	Q-25	30.35	66.93	5000	300	2978
154	Q-26	30.32	66.87	3800	200	2973
155	Q-28	30.32	66.87	3800	200	2973
156	Q-30	30.27	66.97	4300	300	2984
157	Q-32	30.30	66.95	4300	300	2981
158	Q-33	29.78	67.07	4300	300	3006
159	Q-35	30.22	66.78	4300	300	2967
160	Q-36	29.97	66.95	4300	300	2990
161	Qadir Bux Their	28.78	71.40	3800	200	3441
162	Quetta Miri	30.25	66.98	4300	300	2985
163	R.D. 66	29.22	72.87	3800	200	3565
164	Rahmanwali	28.65	71.22	3800	200	3428

Continued on next page

Table S4 – continued from previous page

No.	Site Name	Lat. °N	Long. °E	Age (yr BCE)	$\tilde{\sigma}$ (yr)	Distance from Gesher (km)
165	Rais Sher Mohammad	28.32	66.13	4300	300	2962
166	Rana Ghundai	30.40	68.75	7000	300	3147
167	Rodkan	26.10	64.40	3800	200	2882
168	Sadwala Kanda	28.81	71.11	3800	200	3413
169	Safuwala Ther	28.64	70.98	3800	200	3406
170	Safuwala Three	28.65	71.00	3800	200	3407
171	Safuwala Two	28.64	70.98	3800	200	3406
172	Sahib Khan	30.60	67.05	4300	300	2983
173	Saiyid Maurez Damb	29.43	66.45	5000	300	2958
174	Sala Khan	29.30	66.48	3800	200	2965
175	Santhli Five	23.90	71.50	4000	200	3636
176	Santhli Four	23.90	71.48	4000	200	3634
177	Santhli One	23.90	71.50	4000	200	3636
178	Santhli Six	23.90	71.51	4000	200	3637
179	Santhli Three	23.90	71.48	4000	200	3634
180	Santtili Two	23.90	71.49	4000	200	3635
181	Sanukewala Two	28.86	71.17	3800	200	3417
182	Shahr Sardar	29.45	66.48	3800	200	2960
183	Sheri Khan Tarakai	32.82	70.45	3800	200	3252
184	Sheruwala Three	28.73	71.22	3800	200	3425
185	Sheruwala Two	28.73	71.24	3800	200	3427
186	Shidiwala A	28.78	71.23	3800	200	3425
187	Siah Damb, Surab	28.57	66.18	5000	300	2959
188	Site Near Kuki Damb	28.73	66.35	3800	200	2970
189	Sohniwali	28.75	71.02	3800	200	3406
190	Sohniwali Two	28.75	71.03	3800	200	3407
191	Sorak Damb	27.43	66.47	3800	200	3024
192	Sraduk	27.02	64.18	3800	200	2825
193	SraKala	30.63	66.98	3800	200	2976
194	Sumer Damb	27.16	66.43	3800	200	3030
195	Suneri Damb	27.45	65.75	3800	200	2956
196	SurJangal	30.27	68.50	4300	300	3127
197	Surkh Damb	28.30	66.27	3800	200	2976
198	Tegak	28.32	66.15	4300	300	2964
199	Tharro Hill	24.83	67.82	7000	300	3253
200	Theriwala	29.10	72.81	3800	200	3563
201	Thok Valley One	28.73	66.35	5000	300	2970
202	Thoom Thali	28.77	71.36	3800	200	3437
203	Thoriwala	28.60	71.03	3800	200	3412
204	Toji Damb	28.88	65.67	3800	200	2901
205	Tokaria Timbo	23.47	71.83	4000	200	3686
206	Tor Ghundai	29.75	66.33	3800	200	2938
207	Trillar	29.18	72.21	3800	200	3504
208	Turawewala B	28.78	71.50	3800	200	3450
209	Turawewala C	28.78	71.51	3800	200	3451
210	Turawewali Their	28.78	71.50	3800	200	3450
211	Valwala Two	28.62	70.98	3800	200	3406
212	Valwali	28.63	70.98	3800	200	3406
213	Waddenwali	28.87	71.44	3800	200	3442
214	Wariyal C	29.18	71.91	3800	200	3476
215	Zayak North	27.92	65.90	4300	300	2954
216	Zidi	27.72	66.78	4300	300	3043
217	Zik	26.20	64.78	3800	200	2913

Table S5: The archaeological dates for Iran and Afghanistan [2] (Tepeh Khaleseh is from [57, 58]).

No.	Site Name	Lat. °N	Long. °E	Age (yr BCE)	$\tilde{\sigma}$ (yr)	Distance from Gesher (km)
1	Aq Tappe	37.57	57.42	7000	300	2060
2	Arismān	33.67	52.00	7000	300	1537
3	Čoḡā Āhuwān / Golān	33.38	46.27	7000	300	1005
4	Deh Hajj	33.68	48.88	7000	300	1248
5	Golestān Park	38.08	46.28	7000	300	1147
6	Gŭsa Tappe(Ardabil)	38.25	48.28	7000	300	1311
7	Qomrud	34.73	51.07	7000	300	1456
8	Tepeh Khaleseh	36.19	49.17	6000	300	1312
9	Tappe J̌olbar	38.56	42.32	7000	300	899
10	Tappeh Deh Keir	36.53	54.98	7000	300	1829
11	Tappeh Ozbaki	35.54	50.34	7000	300	1400
12	Tol-e Baši	30.08	52.59	7000	300	1644

References

[1] Possehl GL (1999) *Indus Age: The Beginnings* (Univ Pennsylvania Press, Philadelphia).

[2] Azarnoush M, Helwing B (2005) Recent archaeological research in Iran: Prehistory to Iron Age. *Archaeologische Mitteilungen aus Iran und Turan* 37:189–246.

[3] Böhner U, Schyle D, *Radiocarbon Context Database*, http://context-database.uni-koeln.de, accessed in 2013.

[4] Segal D, Carmi I (2003) Radiocarbon dating. *The Neolithic site of Abu Ghosh. The 1995 Excavations*, IAA Reports 19, eds Khalaily H, Marder O (Israel Antiquities Authority Publ).

[5] Platform for the publication of Neolithic radiocarbon dates. *Ex Oriente,* http://www.exoriente.org/associated_projects/ppnd.php, accessed in 2013.

[6] Rollefson GO (2005) Stone tools from Ayn Jammam, near Ras en-Naqb, Southern Jordan. *Neolithics* 1:17–23.

[7] Fazeli H, Conningham RAE, Batt CM (2004) Cheshmeh-Ali revisited: towards an absolute dating of the Late Neolithic and Chalcolithic of Iran's Tehran Plain, *Iran* 42:13–23.

[8] Riehl S et al (2011) Plant use in three Pre-Pottery Neolithic sites of the northern and eastern Fertile Crescent: a preliminary report. *Vegetation History and Archaeobotany* 21:95–106.

[9] Alizadeh A, Miller NF, Rosen AM, Redding RW (2003) *Excavations at the Prehistoric Mound of Chogha Bonut, Khuzestan, Iran. Seasons 1976/77, 1977/78, and 1996* (Oriental Institute, Univ Chicago, Ill).

[10] Dupree L et al (1972) Prehistoric research in Afghanistan (1959–1966). *Trans Amer Philos Soc* 62:1–84.

[11] Görsdorf J (2000) 14C-Datings of the Es-Sifiya Settlement (Area C). *At the Crossroads. Essays on the Archaeology, History and Current Affairs of the Middle East*, eds Bienert HD, Müller-Neuhof B, Wagner-Lux U, Liedgens I (German Protestant Institute of Archaeology in Amman), pp. 15–19

[12] Simmons AH, Najjar M (2006) Ghwair I: a Neolithic community in Southern Jordan. *J Field Archaeol* 31:77–95.

[13] CZAP : The Central Zagros Archaeological Project, Accessed in 2013 http://www.czap.org/jani

[14] Benz M, Coskun A, Weninger B, Alt K W, Ozkaya V (2010) Stratigraphy and Radiocarbon dates of the PPNA site of Körtik tepe Diyarbakir, *Arkeometri Sonuçlari Toplantisi* vol 26: 81-100

[15] Ozkaya V, Coskun A (2007) Körtik Tepe Kazilari: Erken Neolithik Dönemde Bölgesel Kültürel Ilişkiler Üzerine Bazi Gözlemler, *B. Can and M. Işikli (eds.) Işik D. Arkeologji Yazilari;Atatürk Üniversitesi 50. Kuruluş Yildönümü Arkeoloji Bölümü Armağani;Istanbul* 85-98

[16] Ozkaya V, San O (2007) Körtik Tepe: Bulgular Işiğinda Kültürel Doku Üzerine-Ilk Gözlemler, *Özdoğan M and Başgelen N (eds.) Anadolu'da Uygarliğin Doğuşu ve Avrupa'ya Yayilimi: Türkije'de Neolithik Dönem, Yeni Kazilar, Yeni Bulgular* 21-36

[17] CZAP : The Central Zagros Archaeological Project, Accessed in 2013 http://www.czap.org/sheikh-e-abad

[18] Alizadeh A, Miller N F, Kimiaie M, Mashkour M (2006) The Origins of state organizations in prehistoric highland FARS Southern Iran; Excavations at Tall-e Bakun; Appendix A : Tables 9;10;11, *Oriental Institute Publications* vol 128 ch 11:107-118

[19] Isakov A, Kohl P L, Lamberg-Karlovsky C C, Maddin R (1987) Metallurgical Analysis From Sarazm; Tadjikistan SSR, *Archaeometry* vol 29 num 1: 90-102

[20] Agrawal D P, Krishnamurthy R V, Kusum S (1985) Physical Research Laboratory Radiocarbon Date List V, *Archaeometry* vol 27 num 1: 95-110

[21] Lamberg-Karlovsky C C (1970) Excavations at Tepe Yahya; Iran 1967-1969 PROGRESS REPORT I, *American School of Prehistoric Research and The Asia Institute of Pahlavi University*

[22] Fujii S (2007) Wadi Abu Tulayha and Wadi Ruweishid ash-Sharqi; An investigation of PPNB barrage systems in the Jafr Basin, *Neo-Lithics* vol 2: 6-17

[23] Simmons et al (2001) Wadi Shu'eib; a large Neolithic community in central Jordan: Final report of test investigations *Bulletin of the American Schools of Oriental Research* vol 321: 1-39

[24] Gopher A (1994) Arrowheads of the Neolithlic Levant: A Seriation Analysis, *Eisenbrauns*, Dissertation series:10

[25] Hours F (1994) Atlas des sites du proche orient (14000-5700 BP), *Travaux de la Maison de l'Orient mditerranen* 24

[26] Burleigh R, Ambers J, Matthews K (1982) British Museum natural radiocarbon measurements XV, *Radiocarbon* vol 24 num 3: 262-290

[27] Rollefson G (1998) Expanded Radiocarbon Chronology from Ain Ghazal, *Neo-Lithics* vol 2: 8-10

[28] Cauvin J (1974) Les Débuts de la céramique sur le Moyen-Euphrate : nouveaux documents, *Paléorient* vol 2 num 1: 199-205

[29] Hole F, ed. Aurenche et al (1986) Chronologies in the Iranian Neolithic, Chronologies in the Near East : relative chronologies and absolute chronology 16.000-4.000 B.P. : C.N.R.S. International symposium, Lyon (France), *BAR International Series 379* series(i),24-28 Nov: 353-379

[30] Kozlowski S K (1994) Radiocarbon dates from aceramic Iraq, in Late Quaternary Chronology and paleoclimates of the eastern mediterranean ed. Bar-Yosuf O,Kra R S, *Tucson: Radiocarbon* 255-264

[31] Kozlowski S K (1989) Nemrik 9; a PPN Neolithic site in Northern Iraq, *Paléorient* vol 15 num 1:25-31

[32] Hedges et al (1996) Radiocarbon Dates from the Oxford AMS system: Archaeometry datelist 21, *Archaeometry* vol 38 num 1:181-207

[33] Watkins T, Betts A, Dobney K, Nesbitt M (1995) Qermez Dere; Tel Afar: Interim Report 3, In: Project Paper No. 14 *Department of Archaeology; University of Edinburgh*

[34] Scott EM, Cook GT, Naysmith P (2007) Error and uncertainty in radiocarbon measurements. *Radiocarbon* 49:427–440.

[35] Dolukhanov P, Shukurov A, Gronenborn D, Sokoloff D, Timofeev V, Zaitseva G (2005) The chronology of Neolithic dispersal in Central and Eastern Europe. *J Archaeol Sci* 32:1441–1458.

[36] Aitken M (1990) *Science-based Dating in Archaeology,* p. 98 (Longman, London).

[37] Mazurkevich A, Dolukhanov P, Shukurov A, Zaitseva G (2009) Late Stone – early Bronze age cites in the Western Dvina–Lovat Area. *The East European Plain on the Eve of Agriculture,* British Archaeological Reports International Series 1964, eds Dolukhanov PM, Sarson GR, Shukurov AM (Archaeopress, Oxford), pp 145–153.

[38] Dolukhanov P, Shukurov A (2004) Modelling the Neolithic dispersal in Northern Eurasia. *Documenta Praehistorica* 31:35–47.

[39] Dolukhanov P, Sokoloff D, Shukurov A (2001) Radiocarbon chronology of Upper Palaeolithic sites in Eastern Europe at improved resolution. *J Archaeol Sci* 28:699–712.

[40] Bronk Ramsey C (2009) Bayesian analysis of radiocarbon dates. *Radiocarbon* 51:337–360.

[41] Buck CE, Litton CD, Smith AFM (1992) Calibration of radiocarbon results pertaining to related archaeological events. *J Archaeol Sci* 19:497–512.

[42] Marshall JL (2012) *Missing Links: Demic Diffusion and the Development of Agriculture on the Central Iranian Plateau* (Durham Theses, Durham University, UK. Available at *Durham E-Theses Online:* http://etheses.dur.ac.uk/3547/).

[43] Baggaley AW, Sarson GR, Shukurov A, Boys RJ, Golightly A (2012) Bayesian inference for a wavefront model of the Neolithisation of Europe. *Phys Rev E* 86:016105.

[44] Baggaley AW, Boys RJ, Golightly A, Sarson GR, Shukurov A (2012) Inference for population dynamics in the Neolithic period *Ann Appl Stat* 6:1352–1376.

[45] Draper NR, Smith H (1981) *Applied Regression Analysis* (2nd ed, Wiley Publ, NY).

[46] Leonard T, Hsu JSJ (1999) *Bayesian Methods: An Analysis for Statisticians and Interdisciplinary Researchers* (Cambridge Univ Press, Cambridge, UK).

[47] Akaike H (1978) A Bayesian analysis of the minimum AIC procedure *Ann Inst Statist Math*, A30:9–14.

[48] Burnham KP, Anderson DR (2010) *Model Selection and Multi-Model Inference: A Practical Information-Theoretic Approach,* p. 66 (2nd ed, Springer, NY).

[49] Main IG, Leonard T, Papasouliotis O, Hatton CG, Meredith PG (1999) One slope or two? Detecting statistically significant breaks of slope in geophysical data, with application to fracture scaling relationships *Geophys Res Lett* 26:2801–2804.

[50] Henry DO et. al. (2003) The Early Neolithic Site of Ayn Abu Nukhayla, Southern Jordan *BASOR* 330:1–30.

[51] Marshall JL (2012) Missing Links: Demic Diffusion and the Development of Agriculture on the Central Iranian Plateau *Durham Theses, Durham University* http://etheses.dur.ac.uk/3547/.

[52] Szymczak K, Khudzhanazarov M (2006) *Exploring the Neolithic of the Kyzyk-Kums* p. 26(Institute of Archaeology, Warsaw University).

[53] eds Potts DT, Roustaei K, Petrie CA, Weeks LR (2009) *The Mamasani Archaeologica Project Stage One, A report on first two seasons of teh ICAR- University of Sydney expedition to the Mamasani District, Fars Province, Iran* (Archaeopress, Oxford).

[54] Press WH, Teukolsky SA, Vetterling WT, Flannery BP (2007) *Numerical Recipes: The Art of Scientific Computing* Third edition (Cambridge University Press).

[55] Bernback R, Pollock S, Nashli HF (2008) Rahmatabad : Dating the aceramic Neolithic in Fars province *Neo-Lithics* 1/08:37–39.

[56] Pollard AM, Davoudi H, Mostafapour I, Valipour HR, Nashli HF (2012) A New radiocarbon chronology for the late Neolithic to Iron age in the Qazvin plain, Iran. *Intl. J. Humanities* 19(3):110–151.

[57] Alibaigi S, Khosravi S (2009) Tepeh Khaleseh: a new Neolithic and Palaeolithic site in the Abharrud basin in north-western Iran *Antiquity* 83(319).

[58] Valipour HR, Davoudi H, Sadati JH, Nashli HF (2012) Tepe Khaleseh: archaeological evaluation of a Late Neolithic site in north-western Iran. *Antiquity* 86(331).
